# Species-Specific Retinal Adaptation to Permanent Darkness in *G. charrua* and *O. latipes*

**DOI:** 10.3390/cells15201886

**Published:** 2026-10-15

**Authors:** Laura Herrera-Astorga, Stephanie Silva-Jaureguiberry, Inés Berrosteguieta, Juan Carlos Rosillo, Risa Suzuki, Gero Hofmann, Gabriela Casanova, Joachim Wittbrodt, Anabel Sonia Fernández

**Affiliations:** 1 Laboratorio de Neurobiología Comparada, Departamento de Neurociencias Integrativas y Computacionales, Instituto de Investigaciones Biológicas Clemente Estable (IIBCE), Avenida Italia 3318, Montevideo 11600, Uruguay; lherrera@iibce.edu.uy (L.H.-A.); ssilva@iibce.edu.uy (S.S.-J.); iberrosteguieta@iibce.edu.uy (I.B.); jrosillo@iibce.edu.uy (J.C.R.); 2 Centre for Organismal Studies, Heidelberg University, Im Neuenheimer Feld 230, 69120 Heidelberg, Germany; risa.suzuki@cos.uni-heidelberg.de (R.S.); gero.hofmann@cos.uni-heidelberg.de (G.H.); 3 Heidelberg Biosciences International Graduate School (HBIGS), Im Neuenheimer Feld 501, 69120 Heidelberg, Germany; 4 Unidad de Microscopía Electrónica, Facultad de Ciencias, Universidad de la República, Iguá 4225, Montevideo 11400, Uruguay; casanova@fcien.edu.uy; 5 Laboratorio de Neurociencias, Facultad de Ciencias, Universidad de la República, Iguá 4225, Montevideo 11400, Uruguay

**Keywords:** retinal cell proliferation, adaptive neurogenesis, sensory adaptation, optomotor response

## Abstract

**Highlights:**

**What are the main findings?**

- Prolonged darkness elicits an increase in retinal cell proliferation and visual function changes in *G. charrua* and *O. latipes* (medaka).
- The type of proliferating retinal cells and proliferation dynamics differed between species.

**What are the implications of the main findings?**

- Environmental cues elicit adaptive plasticity responses in the teleost visual system. The nature and functional consequences of these responses are species-specific.
- Comparative approaches on species with divergent adaptive responses provide valuable insights into the mechanisms shaping postnatal retinal neurogenesis and mechanistic range of adaptation to environmental settings.

**Abstract:**

Sustained neurogenesis underlies retinal regeneration in teleosts, but is largely absent in mammals, making comparative studies of closely related fish species a powerful approach to uncover regulatory mechanisms and evolutionary constraints. Environmental cues are likely key regulators, yet their impact on plasticity and function is poorly understood. We examined the impact of prolonged darkness on retinal cell proliferation and visual performance in two closely related teleosts, the annual fish *Garcialebias charrua* and *Oryzias latipes*. EdU/BrdU labeling was used to quantify retinal cell proliferation after defined darkness periods. Visual acuity and contrast sensitivity were assessed via optomotor response assay. Both species exhibited increased cell proliferation in response to darkness; however, the affected retinal layers and dynamics differed. In *G. charrua*, proliferation increased in the inner nuclear layer, with decreased visual acuity and higher contrast sensitivity. *O. latipes* responded to shorter darkness exposure with increased cell proliferation in the outer nuclear layer, decreased acuity and, unlike in annual fish, reduced contrast sensitivity. Our results reveal species-specific recruitment of distinct progenitor/precursor cell populations in the adaptation to changes in the photic environment. Our results highlight the evolutionary relevance of retinal neurogenesis, playing a key role in the adaptation to a changing environment.

## 1. Introduction

Despite the highly conserved anatomy and physiology of the vertebrate retinas [1], comparative studies have evidenced species-specific adaptations related to life history and environmental pressures [2,3,4]. Fish visual systems, in particular, are motors of speciation [5,6] and have been shown to respond to selective pressures imposed by a changing visual environment resulting in fine-tuning to specific light conditions [7,8,9,10,11,12]. Neurogenesis plays a significant role in these plastic phenomena [3,13,14,15,16,17], since the availability of newly generated neurons can contribute to adaptation as the visual requirements of the animal change by modulation of the generation, maturation and integration of new neurons into existing circuits (Reviewed in [1,13,18]).

The teleost retina exhibits persistent neurogenesis throughout life. New cells in the postnatal and adult neural retina of teleosts derive from two main sources: (1) the ciliary marginal zone (CMZ), a neurogenic niche par excellence [19,20,21] and (2) Müller glia [1,22,23]. The CMZ, composed of stem cells and progenitors in the retinal periphery, generates all neural retinal cells, allowing for retinal growth during postnatal life [1,24,25]. On the other hand, Müller glia play a key role in the maintenance of retinal homeostasis and structure [26], as well as in rod production in the adult retina [1,22,23,27,28]. Müller glia give rise to rod precursors, a rapidly dividing population of lineage restricted precursors located in the outer nuclear layer (ONL), that in turn produce rod photoreceptors [21,22,23,28,29,30]. As a response to damage, teleost Müller glia can give rise to all cell types of the neural retina and restore its laminar structure [31], constituting multipotent cells that can be activated in neurogenic processes and thus being the main progenitors in the differentiated retina [21,22].

The contrasting regenerative and neurogenic capacities of teleosts and mammals, have positioned teleosts as major models for understanding the drivers of sustained adult retinal neurogenesis. However, proliferative capacities have been shown to differ markedly even within teleost species [32,33,34,35]. Thus, our understanding of these processes cannot be complete without comparative studies between related species, taking into account the role of their environment, to understand species diversity, functional significance and evolutionary history of adult neurogenesis [20,32]. Thus, fish with particular life cycles provide an exceptional opportunity to understand the plasticity of the visual system as a natural adaptive mechanism [1].

Annual fish of the genus *Garcialebias* (formerly *Austrolebias*) [36] have emerged as an interesting model to study the dynamic adaptation to rapidly changing visual conditions, given their notably short life cycle for a vertebrate and the variable and extreme environmental conditions that characterize their habitat [37,38,39]. These species inhabit shallow freshwater pools that become increasingly turbid and covered by vegetation as summer approaches, resulting in reduced light availability before desiccation. In this context, sensory structures, particularly those related to vision, exhibit plastic responses to changing environmental conditions [40,41,42]. Thus, the annual fish retina constitutes a privileged model for analyzing how environmental factors, such as the availability of light, influence cellular organization, neurogenesis, and neural plasticity.

In fact, permanent darkness exposure can elicit an increase in retinal cell proliferation in the *Garcialebias charrua* retina, where 30 days of darkness increased proliferation in the CMZ and adjacent neural retina [40], while shorter periods of darkness (11 days) showed an effect in the INL [43]. We propose that these responses reflect an increased genesis of rod photoreceptor and the cells that compose their circuits, to improve sensitivity under reduced light conditions. New neurons would be derived from Müller glia, highlighting their ability to not only activate their proliferation to aid regenerative actions, but also as adaptive physiological responses to changes in the visual environment.

*Oryzias latipes* (medaka, Cab strain) constitutes an interesting comparative model, as their Müller glia have been described to possess a constrained potential in regeneration, producing only photoreceptors after damage. Furthermore, both species share the superorder Atherinomorpha [44]. Interestingly, medaka has also been described as a species capable of withstanding a wide range of environmental conditions, including temperature, salinity and water quality [44,45,46,47,48]. Their habitat shows common features to that of annual fishes, as medaka is mainly found in shallow and slow-flowing rice and agricultural channels with abundant plant cover in temperate zones [47,49], but that do not periodically dry out during the summer season.

Comparing species that may differ in their adaptive capacities may help identify the mechanisms and regulators that enable or constrain them, further providing insight into how adaptive or reparative neurogenesis is limited in other vertebrates, including mammals.

Here, to determine how ecological differences and specific adaptations of each species impact the strategies of retinal plasticity related to neurogenesis, we exposed adult *G. charrua* and *O. latipes* to permanent darkness. Exposure to different experimental times in darkness aimed to analyze the temporal progression of possible responses, and whether longer exposure times could result in changes in the type of response (sensory compensations vs. intramodal compensation). Quantification of cell proliferation in the neural retina showed cell proliferation changes differently for each species: *O. latipes* displayed a significantly greater cell proliferation at 14 d of darkness exposure in the ONL, returning to basal levels at longer exposure times. However, *G. charrua* only exhibited greater cell proliferation in the INL, at 33 days of darkness, returning to control levels at longer times. In turn, to determine whether the changes observed in cell proliferation could be correlated with functional changes in vision, we aimed to determine changes in visual acuity and contrast sensitivity through an optomotor response (OMR) assay. This way, we determined that *G. charrua* fish kept in darkness for longer, showed decreased visual acuity and increased contrast sensitivity. Medaka also showed decreased visual acuity when exposed to darkness, while, contrary to annual fish, contrast sensitivity was also decreased.

## 2. Materials and Methods

### 2.1. Animal Capture and Care

#### 2.1.1. *G. charrua*

Male and female adult *G. charrua* fish were collected from the field in La Coronilla, Rocha, Uruguay (33°55′11.4″ S, 53°32′34.7″ W) in October (spring). Fish capture and laboratory maintenance was carried out according to Papa et al. [50]. Briefly, fish were kept in 30 L glass aquaria with constant aeration in pairs or groups of three animals (one male and up to two females). To allow for the display of reproductive behaviors, an artificial plant and coconut fiber was placed in the bottom of the tanks. Water temperature was set to 19 ± 1 °C with constant aeration. The water (reverse-osmosis water with marine salt added to reach 300 ± 50 µS) was partially changed every 5 d. Fish were fed daily with live *Tubifex* sp. All fish were kept under a controlled photoperiod (14 L: 10 D) until exposure to experimental conditions.

#### 2.1.2. *O. latipes*

Adult male and female *O. latipes* (Cab strain) were kept as closed stock held at the Instituto de Investigaciones Biológicas Clemente Estable in 30 L glass aquaria with constant aeration in groups of two or three males, and 5 to 6 females. All tanks were equipped with an artificial plant and a 25 W heater to maintain the temperature at 25 ± 1 °C. Water (reverse-osmosis water with marine salt added to reach 300 ± 50 µS) was partially changed every 3 d. They were fed twice a day with dry food (crushed Vipan Nature, Sera, Heinsberg, Germany), and *Artemia* sp. nauplii every 48 h.

All animal procedures followed NIH guidelines for maintenance and use of laboratory animals and were approved by the local Committee for Animal Care and Research (CEUA, IIBCE, registry number 004/05/2012, date of approval 30 August 2022 and 003/04/2025, date of approval 29 April 2025).

### 2.2. Photic Conditions

**Figure 1:**
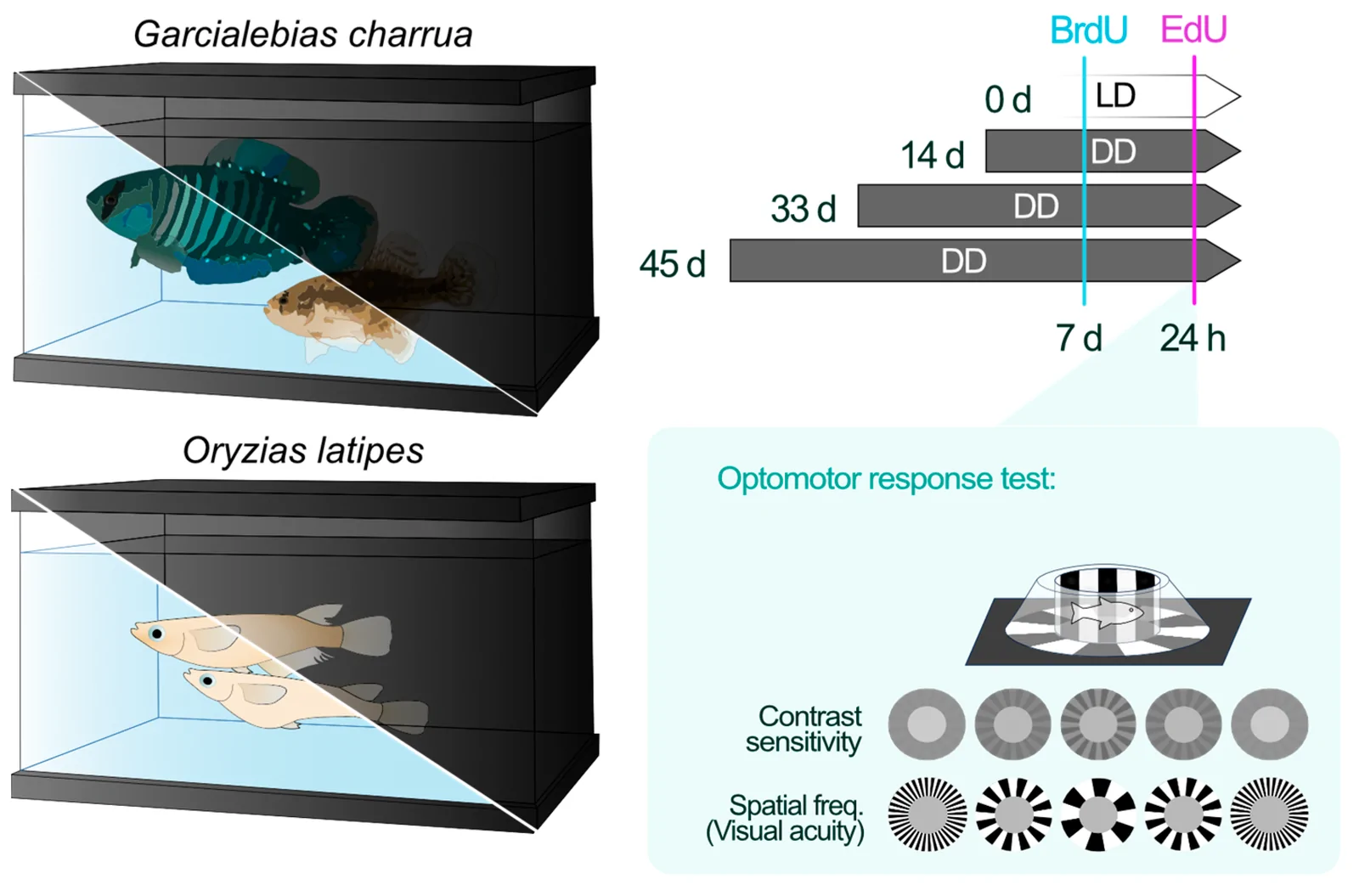
Schematic overview of the experimental design. Specimens were divided into 4 groups (upper right panel): One control group exposed to 14:10 photoperiod (LD) and three darkness groups, exposed to permanent darkness (DD) for 14 d, 33 d, and 45 d. 7 days and 1 day prior to fixation, each specimen was injected intraperitoneally with a dose of BrdU and EdU, respectively. Additionally, the day of fixation, fish were tested in an optomotor response test to determine visual function.

Male and female fish for both species were randomly divided into four experimental conditions (Figure 1).

#### 2.2.1. Permanent Darkness Groups

Specimens of both species were divided into three groups exposed to prolonged darkness conditions with different experimental times: (1) 14 days, (2) 33 days or (3) 45 days.

To achieve darkness conditions, aquaria were placed in shelves with no overhead light, covered with a double layer of black opaque breathable plastic fabric. To further minimize light-exposure, feeding and water changes were performed, switching off the lights in the room and only briefly lifting the dark covering. Light intensity measured at the bottom of the tanks of approx. 0 lux was achieved (measured by HOBO MX2202, Onset Computer Corporation, Bourne, MA, USA).

#### 2.2.2. Control Photoperiod Group (Control)

These specimens were placed in shelves covered with a double layer of black opaque breathable plastic fabric, but with an overhead daylight LED strip to achieve a (14 L:10 D) photoperiod (approx. 460 lux during light period).

### 2.3. Proliferative Marker Injections

Each specimen was removed from their tank, and placed laterally on a wet paper towel, covering the head to immobilize and dim light exposure. The injections were carried out intraperitoneally under stereomicroscope observation using the lowest light setting, with the entire procedure lasting approximately 30 s until return to the aquarium. Dark adapted fish were kept in darkness until injection, and immediately returned to it after. 7 days previous to fixation, each fish was injected intraperitoneally with a single dose (100 mg/kg body weight) of BrdU (Cat. B5002, Sigma-Aldrich, St. Louis, MO, USA) dissolved in 0.9% sodium chloride solution. 24 h previous to fixation, each fish was injected again as described with a single dose (40 mg/kg body weight) of EdU (Cat. NE08701, Biosynth, Compton, UK) dissolved in 0.9% sodium chloride solution.

All injections were performed between 13:00 and 15:00.

### 2.4. Optomotor Response (OMR) Assay

To assess the functional impact in vision, fish were tested in an optomotor response assay to determine changes in visual acuity or contrast sensitivity, using a modification of the setup developed by Suzuki et al. [51] to accommodate adult specimens. Each test arena consisted of a custom-made prism (outer/inner diameter = 20 cm/10 cm, made of polymethyl methacrylate, arena outer/inner diameter = 9 cm/8 cm). For OMR testing the prism was placed on top of an LED Display (Acer K242HQL, 23.6″ and Z-Edge U28I4K, 28″) and trials were recorded using Webcams (Logitech C930e and OBSBOT Meet2, OBSBOT Technology Limited, Kwun Tong, Hong Kong), acquiring videos at a framerate of 30 fps.

Visual stimuli were generated using the Stripe generator software (https://github.com/dkalsan/stripe-generator, accessed on 12 December 2024) by Suzuki et al. [51].

The set-up was placed in a separate testing room covered with Styrofoam and cardboard to minimize sound disturbances and additional light exposure.

Before testing, animals were allowed to acclimate to room light and temperature (24 °C) for 1 h to 1:30 h before trials. After this period, each animal was placed in the testing arena for the contrast sensitivity assay (Figure S1(ai)).

#### 2.4.1. Contrast Sensitivity

All trials started with a 10-min acclimation phase, where each specimen was placed in the test arena, and presented with a stationary homogeneous gray (#B8B8B8) background. After 9:50 min, they were presented with a 10 s stationary version of the first stripe pattern. Each test consisted of moving gray striped stimuli with varying direction and contrast, with constant stripe width (0.2 cpd) and constant speed (3 rpm = 0.31 rad/s = 17.76°/s). After acclimation, each stripe width was presented four times in 30 s intervals, with alternating direction (30 s clockwise (CW) motion, 30 s counterclockwise (CCW), 30 s CW and 30 s CCW). This was followed by a 20 s pause with the homogeneous background grey (#B8B8B8), and 10 s of the stationary version of the next stripe pattern (Figure S1(aii)).

Changes in contrast were set by incrementally darkening and brightening of each stripe pair. The sequence was repeated with progressively higher contrasts: 1% (#818181, #7E7E7E), 2.5% (#838383, #7C7C7C), 5% (#868686, #797979), 10% (#8C8C8C, #737373) and 15% (#939393, #6C6C6C). This was followed by a 60 s pause (50 s background grey + 10 s of the stationary next stripe pattern) and progressively less contrast (15, 10, 5, 2.5 and 1%) (Figure S1(aii)).

After the contrast sensitivity test, animals were removed from the arena and allowed to rest. After 30 min, they were returned to the arena and tested for the visual acuity test.

#### 2.4.2. Visual Acuity Tests (OMR Test with Varying Stripe Width)

All trials started with a 10-min acclimation phase, where each specimen was placed in the test arena, and presented with a stationary homogeneous gray (#B8B8B8) background. After 9:50 min, they were presented with a 10 s stationary version of the first stripe pattern. Each test consisted of moving black and white striped stimuli with varying direction and stripe width, with constant speed (3 rpm = 0.31 rad/s = 17.761692°/s). After acclimation, each stripe width was presented four times in 30 s intervals, with alternating direction (30 s CW, 30 s CCW, 30 s CW and 30 s CCW). This was followed by a 20 s pause with background grey, and 10 s of the stationary version of the next stripe pattern (Figure S1(aiii)).

This sequence was repeated with progressively thicker stripe widths (0.4, 0.6, 0.8, 0.9 and 1 cpd), a 60s pause (50s background grey + 10 s of the stationary next stripe pattern) and progressively thinner stripe widths (*G. charrua*: 0.4, 0.6, 0.8, 0.9 and 1 cpd, *O. latipes*: 0.2, 0.4, 0.6 and 0.8 cpd, cpd were calculated as described in Caves et al. [52]).

#### 2.4.3. Tracking and OMR Analysis: *Oryzias latipes*

The analysis pipeline for the medaka OMR test videos was based on that applied by Suzuki et al. [51].

##### Stripe Timing

The timing of stripe transitions was determined using a custom Python (version 3.11.15) script (Files S1 and S2).

First, the spatial region to be used for stimulus detection was manually annotated, to determine the center of the experimental arena, as well as two crescents of the stimulus visible in the recording. For this, a frame in each video is extracted with OpenCV (version 4.13.0.92), and displayed in an interactive Matplotlib (version 3.11.0) interface (Figure S1(bi)). The resulting arena coordinates and arc parameters are exported as a text file.

Secondly, these coordinates are then used to determine stimulus motion. Normalized grayscale intensities in arc regions were quantified per frame. Stimulus motion was estimated from the mean absolute pixel-intensity difference between each frame and the frame acquired five frames earlier. Timings are then classified into static or moving stimulus blocks. In this case, moving blocks were partitioned into 30-s subphases and assigned clockwise or counterclockwise motions according to the experimental sequence (Figure S1(bii)) and exported as a csv file (File S3).

##### Tracking

Tracking of each individual was automated using a custom Bonsai (Bonsai v2.9.0, https://bonsai-rx.org) workflow. Each OMR trial video was imported to the software, where each arena was cropped using a circular ROI. Then the following nodes were applied: ConvertColor (Rgb2Hsv conversion), HsvThreshold (color threshold was then so that the subject was the biggest segmented ROI), FindContours, BinaryRegionAnalysis, LargestBinaryRegion and Centroid, which were exported as frame-by-frame x,y coordinates in a csv file (Figure S1(ci–cv)).

Custom R (R version 4.4.0, RStudio version 2024.4.1.748) scripts were employed to calculate OMR responses (File S4) [53,54]. Centroid coordinates were used to determine cumulative angular displacement relative to the arena center in 3 s bins (bins were considered invalid if no more than 25% of the frames did not include centroid coordinates or if they were located less than 10 px to the arena center where angular position becomes unstable).

For each valid bin, a response score was calculated as the quotient of net angular displacement divided by the angular displacement of the stimulus [51]. Direction was normalized so that positive values represented movement with the stimulus, such that a response score of 1 matches stimulus angular velocity, and negative values represented opposite direction movement. Bins with response scores ≥ 0.5 were classified as responding with the stimulus (positive) (Video S1).

For each spatial frequency/contrast level, a pooled fraction of positive response bins was calculated. Visual acuity/contrast threshold was defined as the highest cpd or lowest contrast level, respectively, when this fraction was ≥0.5.

ChatGPT 5.5 and 5.6 was employed for assistance in the generation of the code and its documentation.

#### 2.4.4. Tracking and OMR Analysis: *Garcialebias charrua*

The *G. charrua* individuals displayed different behaviors from that of medaka, making the previously used automatic analysis pipeline not adequate, as *G. charrua* fish showed markedly longer response latencies, shorter stimulus driven following, interrupted with periods of frequent erratic or possibly aggressive movements towards reflections (Video S2). We therefore used an alternative approach to quantify the optomotor response in this species. For this, videos were manually analyzed assigning the following scoring system, modified from that described by Caves et al. [52]. A body rotation (by either swimming or pivoting) of at least 180° in the direction and at similar speed to that of the stimulus was scored as 1 (Video S3), whilst a turn between 90° and 180° a score of 0.5 (intermediate response). These scores were then averaged for each spatial frequency/contrast value.

Thresholds were calculated as the highest spatial frequency/contrast value where the mean response score was ≥0.375 (such that at least 75% of the testing showed an intermediate response). Animals that did not show any following at any contrast/frequency value were excluded.

#### 2.4.5. OMR Data Statistical Analysis

OMR data between darkness exposure groups was statistically analyzed through Kruskal-Wallis test followed by a post hoc Dunn’s test (with a Benjamini-Hochberg correction). Each data point corresponds to the values calculated for one specimen.

### 2.5. Specimen Fixation

After the completion of each specimen’s trials, they were fixed as follows.

Fish were deeply anesthetized by adding a 1:1000 *v*/*v* solution of 10% Eugenol (Sigma-Aldrich) to aquarium water until opercular movement cessation. They were then intracardially perfused according to Fernández et al. [55] with saline solution, followed by 10% paraformaldehyde (PFA) (Cat. 158127, Sigma-Aldrich) in 0.1 M phosphate buffer (PB; pH 7.2). After perfusion, fish were postfixed in 10% PFA for another 24 h. Eyes were then removed and washed in PB until further processing.

### 2.6. Tissue Processing, Immunolabeling and Staining

#### 2.6.1. Vibratome Sectioning

Right eyes were embedded in 0.5% gelatin (Cat. 0639, Polyscience, Warrington, PA, USA)–30% Bovine Serum Albumin (BSA, Cat. A9418, Sigma-Aldrich) crosslinked with 1.3% glutaraldehyde (Cat. 4157.1, Carl Roth) and sectioned para-sagittally (60 µm thickness) on a vibratome (Vibratome S100, Leica, Buffalo Grove, IL, USA). The blocks were trimmed to maintain dorso-ventral orientation.

#### 2.6.2. Free Floating Immunolabeling

Sections were incubated at 60 °C for 15 min in 150 mM Tris-HCl pH 9.0. After PBS washes (3 × 10 min), retinal sections were incubated in 1.7 N HCl, 0.5% Triton X-100 (Cat. T9284, Sigma-Aldrich) solution in PBS at RT for 45 min and washed in PBS again (3 × 10 min). To minimize background noise, sections were incubated for 1 h in a blocking solution composed of 1% BSA, 10% Normal Goat Serum (NGS, Cat. G6767–100 mL, Sigma-Aldrich) in PTW (1× PBS, Cat 100110023, Thermo Scientific, Eugene, OR, USA, 0.05% Tween20, Cat. P7949, Sigma Aldrich). Then, sections were incubated with anti-BrdU primary antibodies (1:100 in PTW, Cat. ab6326, Abcam, Rat, monoclonal, RRID:AB_305426) for 48 h at 4 °C. After this, they were washed again (3×) with PBS and incubated in anti-rat secondary antibodies (1:750, Cat. 112-505-143, Goat, Polyclonal, Jackson ImmunoResearch Laboratories, Ely, UK) and 5 μg/mL 4′,6-diamidino-2-phenylindole (DAPI, Cat. D9564-10MG, Sigma-Aldrich) for 1:30 h at room temperature.

#### 2.6.3. EdU Staining

After antibody staining, sections were washed (3 × 10 min in PBS) and incubated in EdU labeling solution for 1 h at RT: 50 mM Ascorbic Acid (Cat. A92902-100G, Sigma-Aldrich), 0.1 M Tris (Cat. 4855.2, Carl Roth), 0.1 μM Alexa Fluor™ 647 Azid (Cat. A10277, Thermo Fisher Scientific), 0.1 μM Copper(II) sulfate (Cat. C7631-1KG, Sigma-Aldrich) [20]. Sections were then washed 3 × 10 min in PBS, and mounted for confocal microscopy using 60% glycerol in PBS.

#### 2.6.4. TUNEL Staining

An additional 3 sections per sample were stained to assess cell death and cell density. Sections were permeabilized by incubation in 0.5% Triton X–100 (Cat. T9284–500 mL, Sigma-Aldrich), 0.1% sodium citrate incubation (Cat. 71405–1 kg, Sigma-Aldrich) solution for 30 min at RT. After 3 × 10 min PBS washes, sections were incubated in the In Situ Cell Death Detection Kit (Cat. 12156792910, Roche, Sigma-Aldrich) following the manufacturer’s instructions. Briefly, after permeabilization, sections were incubated in 1:10 Enzyme Solution, 9:10 Label Solution for 60 min at 37 °C. After PBS washing (3 × 10 min) they were incubated in 6 μg/mL DAPI in PTW and washed 3 × 10 min in PBS and mounted as previously described.

#### 2.6.5. Imaging

Up to three sections closest to the exit point of the optic nerve in each specimen were selected for imaging for BrdU+ and EdU+ quantification. Confocal image stacks (51 μm) of the entire length of the retina were obtained, imaging every 3 μm, using a Leica TCS Sp8 Dmi8 inverted confocal microscope (20× oil immersion objective).

For TUNEL and cell density quantification, up to three sections closest to the exit point of the optic nerve in each specimen were used to obtain confocal image stacks (50 μm) of the dorsal and ventral CMZ and differentiated neural retina were obtained, imaging every 2 μm, using a Leica TCS Sp8 Dmi8 inverted confocal microscope (63× oil immersion objective).

### 2.7. Quantitation and Statistical Analysis

Statistical tests were performed using PAST (V5.2) [56] statistical software.

#### 2.7.1. Proliferative Nuclei Data

For each retinal section labeled, BrdU+, EdU+ and double labeled nuclei were manually quantified along the retina in the ONL and INL, using the Cell Counter Plugin in the FIJI software (v2.16.0/1.54p) [57]. Density was estimated as nuclei counts per mm of retinal length (measured at the base of the INL) for each retinal section. Each section was used as a data point. Statistical differences between experimental groups were analyzed using a Kruskal-Wallis test, followed by Dunn’s post-hoc test with Bonferroni corrections for multiple comparisons.

#### 2.7.2. Cell Density and TUNEL+ Nuclei

To estimate cell density, DAPI stained nuclei were quantified in a region of interest of 100 μm [58] of retinal length in the CMZ adjacent regions and ventral and dorsal center regions (NR), using a single z stack slice. TUNEL+ cells were quantified along the entire z-stack. The dorsal and ventral cell density values for each of the regions (either CMZ or NR) were averaged per retinal section. Since TUNEL+ presence was sporadic, the ventral and dorsal values per retinal sections and region were summed. Statistical differences between experimental groups were analyzed using a Kruskal-Wallis test, followed by Dunn’s post-hoc test with Bonferroni corrections for multiple comparisons.

Figures were prepared using FIJI (v2.16.0/1.54p) [57] Affinity Designer 2, version 2.5.3 (2023).

## 3. Results

### 3.1. Cell Proliferation in Control Photoperiod and Permanent Darkness Conditions

#### 3.1.1. Cell Proliferation in Control Photoperiod Along the Neural Retina

We first determined baseline differences between *G. charrua* and *O. latipes* retinal cell proliferation under controlled photoperiod conditions. Administration of two different proliferation markers (EdU and BrdU) sequentially was aimed at determining the dynamics and location of newly generated cells (Figure 1): EdU+ cells (short chase period) were likely produced in that location, BrdU+ (7 d chase) cells may have undergone migratory/differentiation processes, while double labeled cells (EdU+/BrdU+) likely divided at least twice, indicative of possible progenitor/precursor cells [40,59].

**Figure 2:**
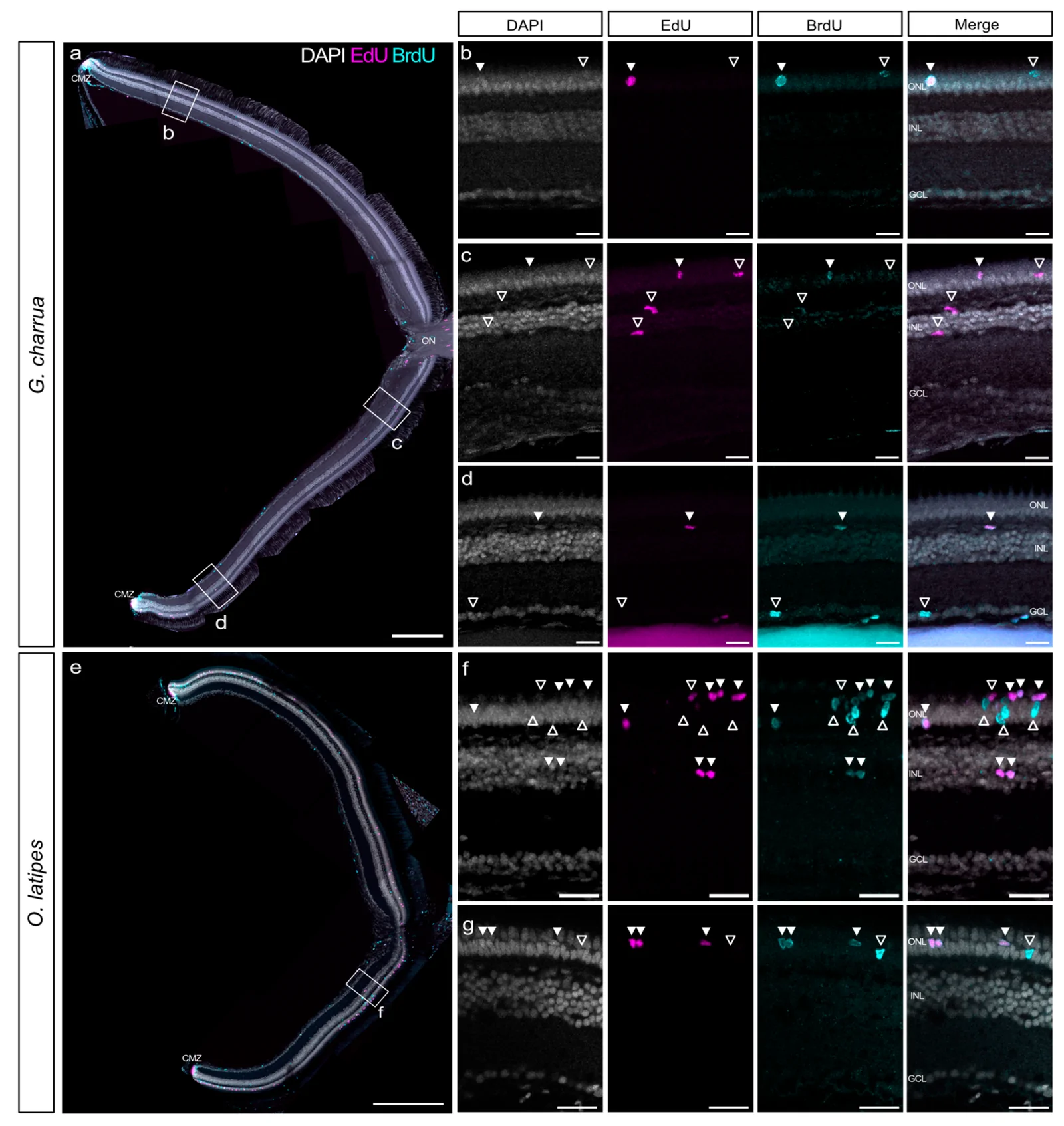
Cell proliferation in the *G. charrua* and *O. latipes* adult retina under control photoperiod conditions. (**a**,**e**) Panoramic view of a complete retinal section of *G. charrua* (**a**) and *O. latipes* (**e**) showing cell proliferation labeled with EdU (magenta) and BrdU (cyan) (Maximum intensity projections, 50 μm z-stack). Boxes indicate the regions displayed in (**b**–**d**,**f**). Scale bars: 250 μm. (**b**–**d**,**f**) Enlarged view of the regions indicated in (**a**,**e**). Double labeled cells with EdU and BrdU are indicated with filled arrowheads. Those labeled with only one marker are indicated with open arrowheads (maximum intensity projections, 30 μm z-stack). (**g**) Higher magnification image of the neural retina, showing double labeled cells with EdU and BrdU in the ONL. Filled arrowheads indicate double labeling, open arrowheads show labeling with only one marker. Abbreviations: CMZ: Ciliary marginal zone, GCL: Ganglion cell layer, INL: Inner nuclear layer, ONL: Outer nuclear layer.

Both species displayed positively labeled nuclei along the entire length of the neural retina, which was more concentrated in the CMZ and adjacent NR, the majority of which were found in the ONL, but also in smaller numbers in the INL and sparsely in the GCL (Figure 2).

Proliferating cells in the ONL displayed differential locations in the apicobasal axis. BrdU+ cells were mainly distributed in the basal regions of the ONL among the rod photoreceptor layer, suggesting a rod photoreceptor fate of the newly generated cells. EdU+ and EdU+/BrdU+ cells were located more apically in an intermediate position between cone and rod nuclei (Figure 2b,f,g), consistent with rod precursor identity [30,60]. In the case of the INL, BrdU+, EdU+ or BrdU+/EdU+ were mainly located in the apical most and basal most regions of the INL (Figure 2c–f), likely due to actively proliferating Müller glia and their progeny.

#### 3.1.2. *O. latipes* ONL Cell Proliferation, but Not INL, Is Higher than That of *G. charrua*

**Figure 3:**
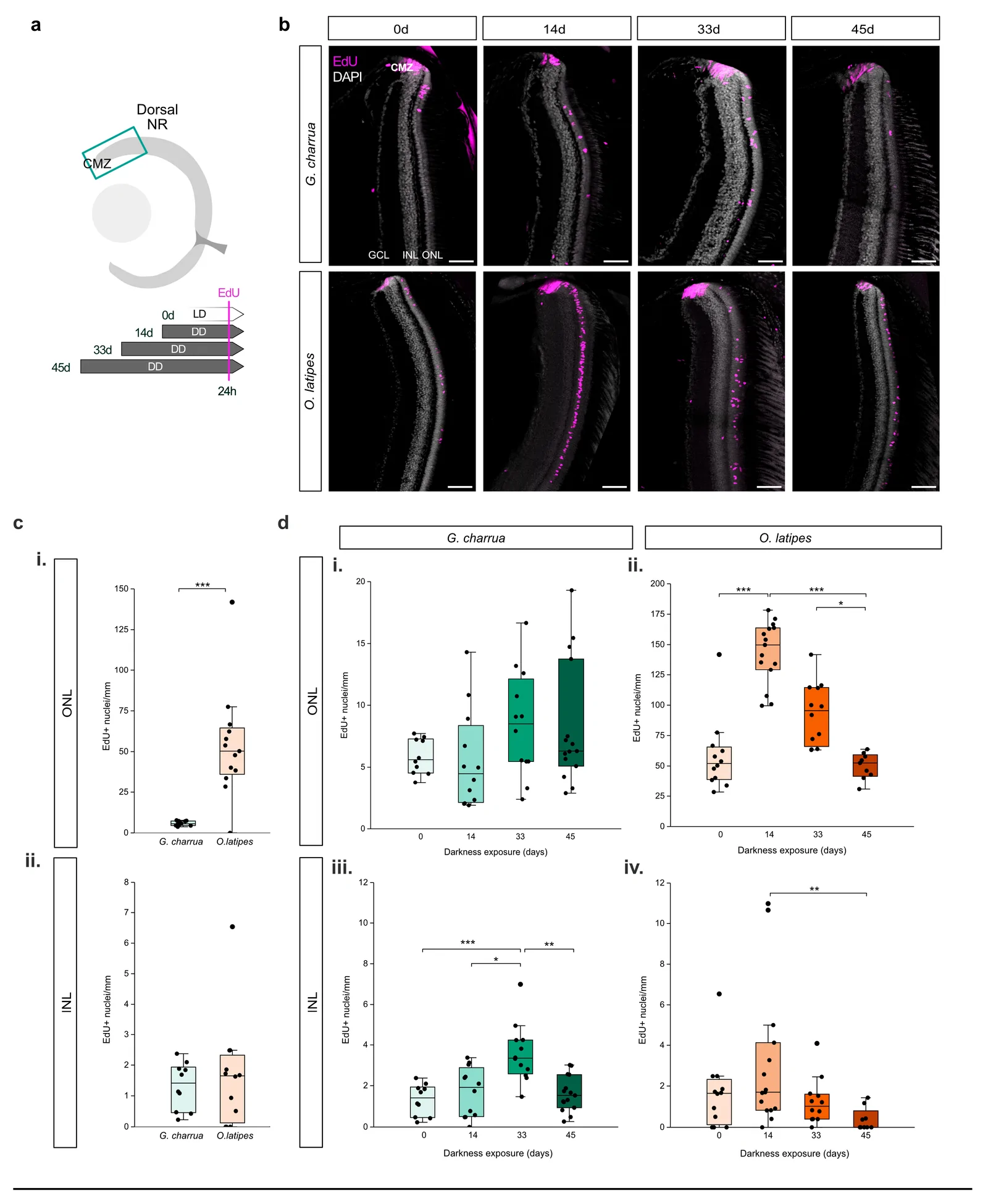
Inter-species retinal cell proliferation differences after exposure to darkness in the *G. charrua* (green) and *O. latipes* (orange) retina. (**a**) Schematic representation of the area depicted in (**b**). Experimental design for the darkness exposure groups. (**b**) Maximum intensity projections of the CMZ and dorsal neural retina depicting the EdU+ nuclei (magenta) for each species at different darkness exposure times (50 μm z-stack). Scale bars: 50 μm. (**c**) Number of EdU+ nuclei per mm of retinal length for the ONL and INL of each species (*G. charrua*—n: 10/4, *O. latipes*—n: 12/5). (**d**) Number of EdU+ nuclei per mm of retinal length for for the ONL and INL of each species under darkness exposure periods (*G. charrua*—0 d n: 10/4; 14 d n: 12/4; 33 d n; 12/4, 45 d n: 15/5; *O. latipes*—0 d n: 12/5; 14 d n: 15/7; 33 d n: 12/5; 45 d n: 9/4). Box plots show median, and 25th and 75th percentiles, whiskers extend to the most extreme data points within 1.5 × IQR of the lower and upper quartiles. For each graph, sample numbers are expressed as the number of retinal sections/number of specimens. Significant differences were determined via Kruskal-Wallis and Dunn test (Bonferroni correction), significance is depicted as follows: * *p* < 0.05, ** *p* < 0.01, *** *p* < 0.001. Abbreviations: CMZ: Ciliary marginal zone, DD: Sustained darkness group, GCL: Ganglion cell layer, INL: Inner nuclear layer, LD: Control photoperiod group, NR: Neural retina, ONL: Outer nuclear layer.

Quantitation of the proliferative cells along the retina of both species revealed differences when comparing control photoperiod conditions. Strikingly, in control conditions, the number of EdU+ and BrdU+ proliferative nuclei were up to an order of magnitude higher in medaka ONL than in the annual fish (EdU: Mann Whitney test U = 10, z = 3.3799, *p* = 0.0007, r = 0.72, BrdU: U = 0, z = 3.9233, *p* = 0.00009, r = 0.83; EdU/BrdU: U = 0, z = 3.80, *p* = 0.00014, r = 0.81) (Figure 3(ci), Figures S2(ci) and S3(ci)). However, this was not the case when comparing the INL (EdU: Mann Whitney test U = 59, z = 0.033, *p* = 0.97, r = 0.007; BrdU: U = 59, z = 0.033, *p* = 0.97, r = 0.007; EdU/BrdU: U = 59, z = 0.033, *p* = 0.97, r = 0.007) (Figure 3(cii), Figures S2(cii) and S3(cii)).

#### 3.1.3. Cell Proliferation Is Increased Species-Specifically After Darkness Exposure

Having detected the presence and location of actively proliferating cells in the adult retina of both species, we sought to determine whether photic conditions can affect retinal cell proliferation. For this, the number of proliferative cells was quantified along the retina in both species for the INL and ONL after different darkness exposure times.

In *G. charrua*, significant differences were detected in the number of EdU+ cells in the INL along exposure times to darkness. Cell proliferation showed gradually increasing median values, with statistical differences at 33 days of exposure, falling again to control levels after the longest (45 d) exposure times (Figure 3(di), Table S1). However, no significant differences were detected when comparing ONL cell proliferation (Figure 3(diii), Table S1).

When analyzing cell proliferation of the medaka retina, significant differences were only detected in the ONL. Here, cell proliferation displayed significantly higher EdU+, BrdU+ and double labeled nuclei at 14 d of exposure, decreasing again to comparable values to that of controls after 45 d (Figure 3(dii), Figures S2(dii) and S3(dii) and Table S1). However, no significant changes were observed in the *O. latipes* INL (Figure 3(div), Figures S2(div) and S3(div) and Table S1).

#### 3.1.4. Higher Cell Proliferation Was Not Related to Increased Cell Death or Cell Density Changes

To determine whether exposure to darkness and increased cell proliferation could be related to excessive cell density or increased cell death, TUNEL staining was combined with nuclear staining to determine the presence of apoptotic nuclei and measure nuclei number in the INL and ONL.

TUNEL+ nuclei were only sporadically observed in the differentiated layers of the retina, mainly in the regions adjacent to the CMZ (Figure S4b) (median values in all groups and regions between 0 and 1). In *G. charrua*, significant differences were only observed in the differentiated NR in the ONL of *G. charrua* between control photoperiod (0 [0–0]) and 45 days of exposure to darkness (0.5 [0–1]), and not in the INL (Figure S4d), supporting that cell death did not show the same patterns or layers as the increases in cell proliferation.

In medaka, significant differences were only detected in the NR ONL between control (0 [0–0]) and 14 days of darkness (0 [0–1]) (Figure S4f). Statistical significance was not reached for the ONL in the CMZ-adjacent retina of medaka, but comparison of the values for control (0 [0–0]) and 14d of darkness (1 [0–2]) showed a *p* value of 0.073 (Dunn’s post hoc, Bonferroni corrected *p*). No significant differences were detected in the INL in either region (Figure S4). Here, in the case of medaka, TUNEL+ count increased at the 14 d timepoint, as did cell proliferation. Thus, higher cell death leading to increased cell proliferation cannot be discarded. However, given that cell proliferation presented values at least one to two orders of magnitude higher than TUNEL+ count, we conclude that this small increase in cell death is likely not a significant factor shaping this process.

Next, cell numbers in each layer were assessed for each species and darkness exposure group. Cell densities in control conditions differed between species, where medaka retinae showed a higher number of cells per 100 μm retinal length (Figure S5), likely related to a smaller nucleus size (Figure S6). Comparison of cell densities in the ONL and INL did not reveal significant differences between control and darkness exposed groups (Figure S5).

### 3.2. Optomotor Response Assay: Functional Consequences of Darkness Exposure

After detecting these changes in response to darkness at the tissue level, to assess whether prolonged darkness exposure could also have functional impacts on vision, fish were tested in an optomotor response assay to determine changes in visual acuity or contrast sensitivity.

#### 3.2.1. Spatial Acuity Is Decreased After Exposure to Darkness

**Figure 4:**
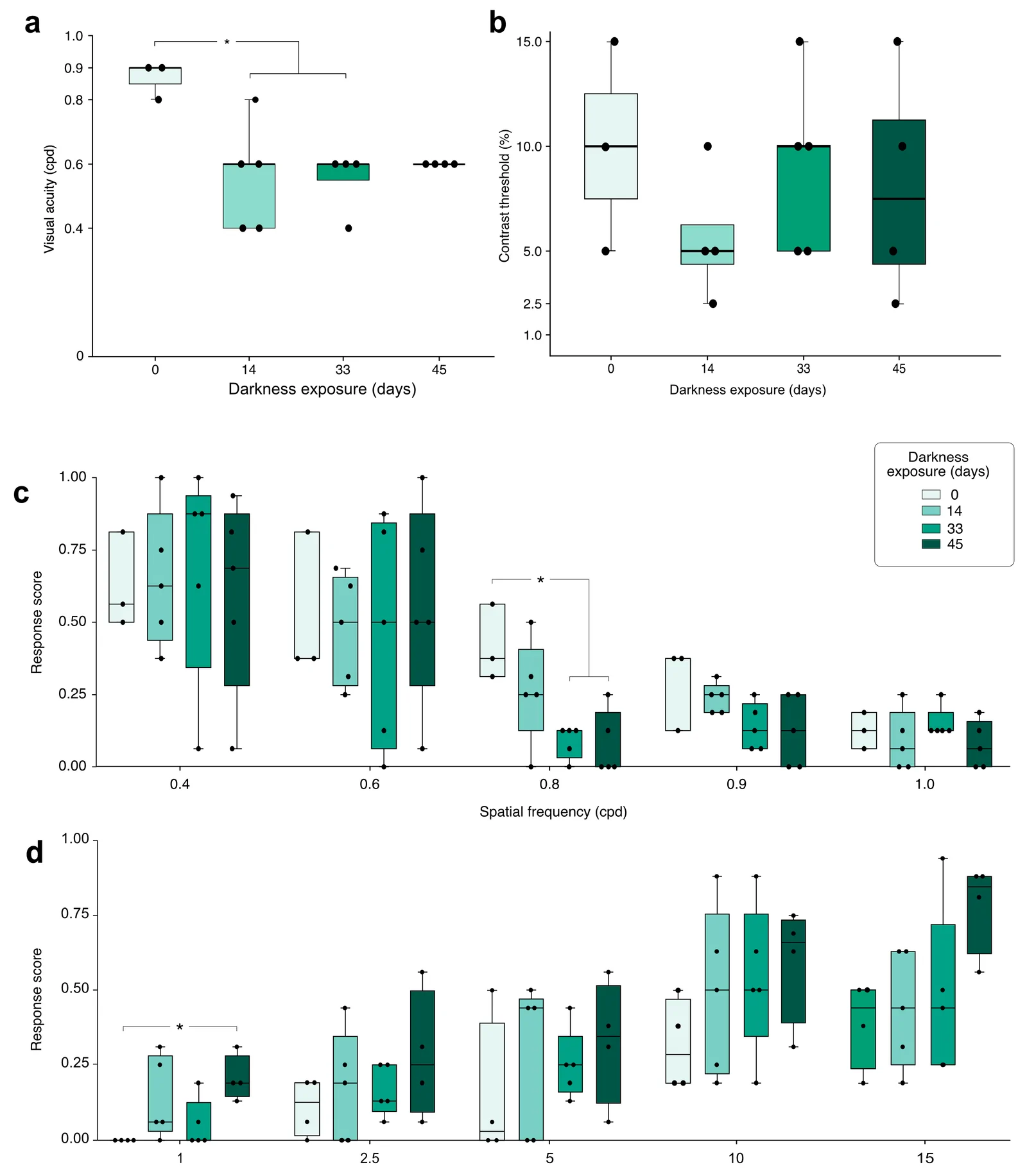
Contrast sensitivity and visual acuity optomotor response tests for *G. charrua* kept under prolonged darkness. (**a**,**b**) Visual acuity (**a**) and contrast sensitivity (**b**) thresholds across darkness exposure times. Threshold values were calculated as the highest spatial frequency (**a**) or lowest contrast level (**b**) at which the mean response value was ≥0.375. (0 d n = 3; 14 d n = 4; 33 d n = 5, 45 d n = 4, animals not displaying a mean response value above 0.375 at any point of the test are not shown in the boxplot). (**c**,**d**) Mean response score for each spatial frequency (**c**) or contrast percentage (**d**). Each value was calculated as: a body rotation of at least 180° in the direction of stimulus is scored as 1, whilst a turn between 90° and 180° a score of 0.5. ((**c**) 0 d n = 3; 14 d n = 5; 33 d n = 5, 45 d n = 4; (**d**) 0 d n = 4; 14 d n = 5; 33 d n = 5, 45 d n = 4). Box plots show median, and 25th and 75th percentiles, whiskers extend to the most extreme data points within 1.5 × IQR of the lower and upper quartiles. Each data point corresponds to one specimen. Significant differences were determined via Kruskal-Wallis and Dunn tests (Benjamini-Hochberg correction). Abbreviations: cpd: cycles per degree. Box plots show median, and 25th and 75th percentiles, whiskers show maximum and minimum data points, Significance is depicted as follows: * *p* < 0.05, ** *p* < 0.01, *** *p* < 0.001.

To determine visual acuity changes, fish were tested using black and white striped stimuli with varying spatial frequencies. The responses of each species were first used to determine changes in the threshold values as a proxy of visual acuity. This way, we detected significant differences in the threshold values in *G. charrua*: control fish displayed a higher threshold, than those exposed to 14 d and 33 d of darkness, i.e., being able to respond to thinner stripes (Figure 4a). For the case of 45 d, even though median values coincided with those of 14 and 33 d, only a tendency was detected (*p* = 0.07). When analyzing the response score across all spatial frequencies, significant differences were observed at intermediate values tested. At 0.8 cpd, fish exposed to longer darkness times (33 d and 45 d) showed significantly lower response values than control photoperiod fish (Figure 4c).

**Figure 5:**
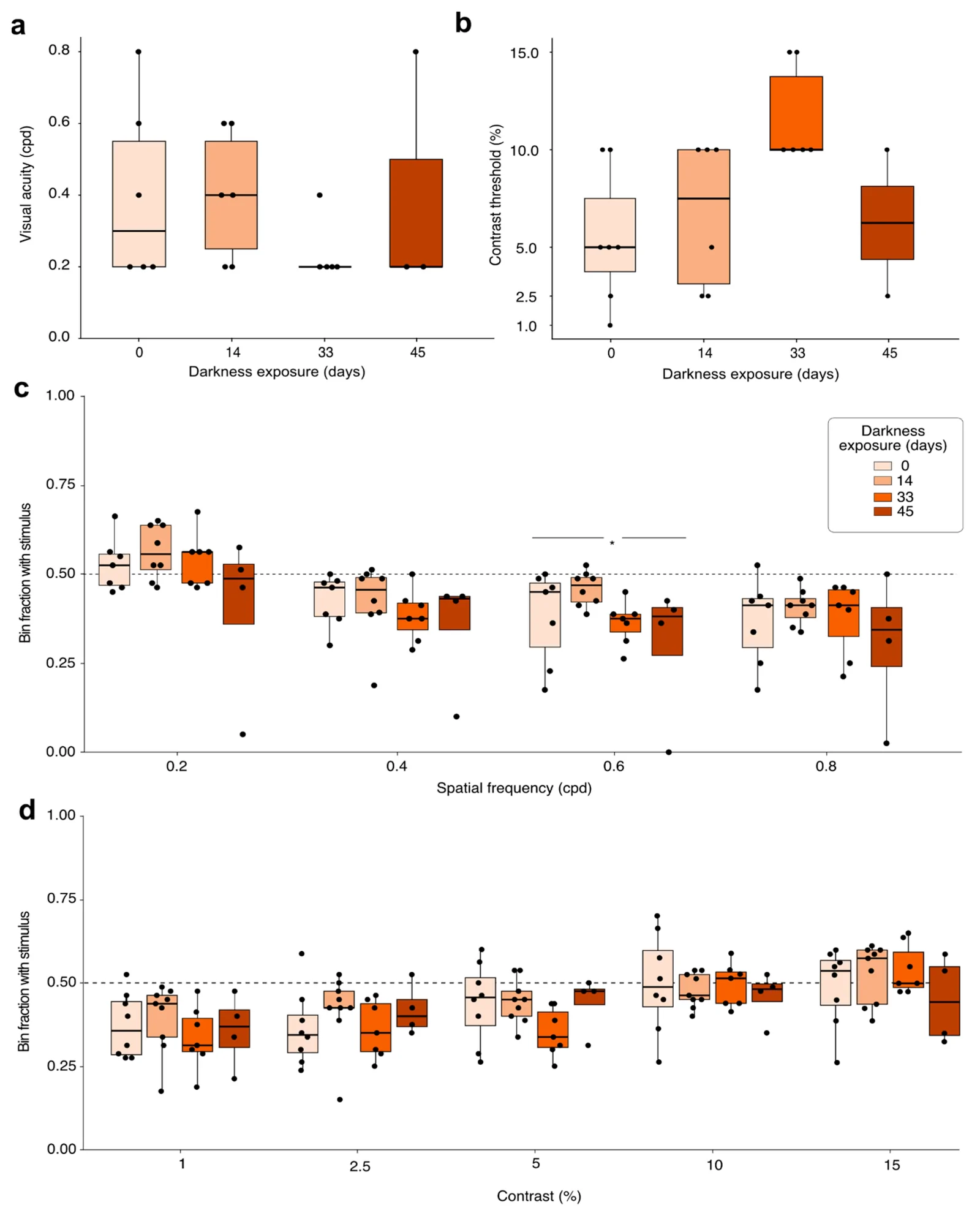
Contrast sensitivity and visual acuity optomotor response tests for *O. latipes* kept under prolonged darkness. (**a**,**b**) Visual acuity (**a**) and contrast sensitivity (**b**) thresholds across darkness exposure times. Threshold values were calculated as the highest spatial frequency (**a**) or lowest contrast level (**b**) at which the bin (3 s intervals) fraction positive to stimulus movement was ≥0.5. (**a**) 0 d n = 6; 14 d n = 6; 33 d n = 5, 45 d n = 3; (**b**) 0 d n = 7; 14 d n = 6; 33 d n = 6, 45 d n = 2; animals not displaying a mean response value above 0.5 at any point of the test are not shown in the boxplot. (**c**,**d**) Fraction of bins (3 s) for which the specimen responded with stimulus for each spatial frequency (**c**) or contrast percentage (**d**). ((**c**) 0 d n = 7; 14 d n = 8; 33 d n = 7, 45 d n = 4; (**d**) 0 d n = 8; 14 d n = 9; 33 d n = 7, 45 d n = 4). Box plots show median, and 25th and 75th percentiles, whiskers extend to the most extreme data points within 1.5 × IQR of the lower and upper quartiles. Each data point corresponds to one specimen. Significant differences were determined via Kruskal-Wallis and Dunn tests (Benjamini-Hochberg correction). Abbreviations: cpd: cycles per degree. Box plots show median, and 25th and 75th percentiles, whiskers show maximum and minimum data points. Significance is depicted as follows: * *p* < 0.05, ** *p* < 0.01, *** *p* < 0.001.

For medaka, no significant differences were observed in visual acuity thresholds (Figure 5a), however, a more detailed analysis of the response along spatial frequencies, detected significant differences at intermediate values as well, at 0.6 cpd (H = 7.82, *p* = 0.0499, η2 = 0.219) (Figure 5c). However, when analyzing which groups differed, Dunn’s post hoc tests could not resolve the differences, detecting only tendencies towards decreased responses at 33 d and 45 d (14 d vs. 33 d: *p* = 0.076; 14 d vs. 45 d: *p* = 0.097).

#### 3.2.2. Contrast Sensitivity Is Increased in *G. charrua* After Exposure to Darkness, but Decreased in *O. latipes*

In the case of the contrast sensitivity threshold (Figure 4b), no significant differences were detected in *G. charrua* between groups. However, when the response scores are analyzed more carefully along the different contrast values, a significant difference was detected at the lowest contrast level (1%) (Kruskall-Wallis: H = 9.20, *p* = 0.027, η2 = 0.443), where contrast sensitivity values were significantly higher at 45 d than in controls (Dunn’s post hoc *p* = 0.037) (Figure 4c). At the highest contrast level, (15%) a strong effect was also detected where 45 d specimens displayed higher median values, however no statistical differences were detected (H = 7.209, *p* = 0.06, η2 = 0.301) (Figure 4c).

Interestingly, a strong effect (H = 7.77, *p* = 0.051, η2 = 0.281) was observed in medaka in the contrast sensitivity threshold, but contrary to annual fish, they displayed a tendency to a decreased sensitivity at 33 d (Dunn’s post hoc *p* = 0.0518), later returning to values similar to control levels (Figure 5b).

## 4. Discussion

This work focused on determining the role of retinal cell proliferation, and consequently of retinal neurogenesis, in adaptation to a dynamically changing environment. Here we compared two related teleost species (*Garcialebias charrua* and *Oryzias latipes*) and evaluated the temporal course of the effects of prolonged darkness. We examined how cell proliferation and visual performance of the retina, a highly plastic organ, are shaped by prolonged darkness as a model for the dynamic environmental change in the life cycle of annual killifish.

We show how prolonged darkness indeed enhanced retinal cell proliferation in both annual fish as well as in medaka. This was accompanied by changes in visual function as assessed by optomotor response assays. In both species, visual changes followed the proliferative response after a latency period, possibly reflecting the time that newly generated cells require to differentiate and integrate into existing circuits before having scorable functional consequences. However, the affected retinal layers, temporal dynamics, and direction of the visual changes strikingly differed between species.

When visual acuity was assessed, both species showed strong effects towards a decrease after the exposure to longer times of darkness. This common response likely reflects a conserved functional trade-off, as has been shown for species adapted to low-light habitats, displaying eye adaptations that prioritize light sensitivity over maintaining visual acuity [61,62]. Interestingly, the assessment of contrast sensitivity revealed a divergent response between species. While medaka showed decreased contrast sensitivity after 33 d of darkness exposure returning to control levels at later timepoints, *G. charrua* showed improved contrast sensitivity at the latest time points. This indicates that each species employs a distinct adaptive strategy activating different precursor or progenitor cell populations. Given their distinct cellular potentials, recruitment of different progenitor or precursor populations may influence not only the types of daughter cells generated, i.e., neurons in retinal circuits or exclusively rod photoreceptors, but also the timing of the response, with more committed precursor cells producing differentiated cells more rapidly reducing the latency in the response. This would lead to functional changes better suited for the specific ecological conditions experienced by each species, but also to the natural progression of those conditions over time, as may occur in *G. charrua* where environmental changes are gradual and sustained, whilst medaka may experience low light conditions transiently.

In medaka, only cells in the ONL, but not in the INL, sharply increased proliferation already at the shortest exposure to constant darkness (14 d) analyzed. This aligns well with a similar transient response reported for zebrafish, where eight days of sustained darkness activated ONL rod precursors, but not INL resident Müller glia, followed by a return to control levels at later time points [63]. A rapid response of this kind likely relies on a population of precursor cells (derived from Müller glia prior) readily available in the ONL, as present in the resting retina of adult medaka. These could be re-activated, synchronized or upregulated to facilitate a rapid cellular (neurogenic) response to environmental changes, including changes in light levels. The resulting increase in rod photoreceptors may compensate for reduced light availability and thus may allow maintaining retinal sensitivity by more photoreceptors or increasing receptive field size, thereby supporting vision under low-light conditions.

In *G. charrua*, adaptation to low-light conditions modeled by complete darkness apparently follows a different path. While activation of cell proliferation is the common theme, this occurs at a later time point and in different cell types of the INL in *G. charrua*. On the functional level, assessed by an OMR assay, we detected improved contrast sensitivity, particularly at the lowest contrast levels, indicating an improved visual performance. We had previously suggested that Müller glia not only acts as a multipotent progenitor in regeneration, but also does so in adaptive responses [43]. Thus, activation of Müller glia enables the production of additional neural elements to adjust the retinal circuits, to enhance and fine-tune vision in low light conditions.

The divergent responses observed comparing the two species are a reflection of different adaptive strategies shaped by the context of their ecological niches. The response observed in the annual fish represents a specialized strategy associated with the extreme and highly variable conditions of their natural habitat. A proliferative response targeted to the INL contributes to the maintenance of a high visual performance in a growingly turbid temporary pond [64]. This provides a selective advantage for *G. charrua*, especially as vision plays a key role in courtship, agonistic behavior or hunting [41,42]. Even though naturally living close to the surface of the shallow waters in rice paddies and associated streams, an adaptive proliferative response to the exposure of darkness was also observed in medaka. In contrast to *G. charrua*, the cells of the ONL were displaying a proliferative response, resembling what has been previously described for zebrafish [63]. Fish living in saturated lighting conditions still show a general response to low light environments, in which the proliferative activity of rod precursor cells is regulated as a compensatory mechanism, even if this may not represent a common ecological challenge in their natural habitats [47,49,65]. This highlights a remarkable plasticity and adaptability of the visual system, prepared to respond to change that does not naturally occur.

These responses are likely a part of a broader pattern of visual plasticity that also involves the ciliary marginal zone, as well as higher-order processing structures and other sensory modalities. We have previously shown that sustained darkness induced increased cell proliferation of the CMZ and of all nuclear layers of the adjacent neural retina [40]. More recently, analysis of long-term exposure to darkness in *Garcialebias reicherti* females revealed olfaction to be preferentially used over vision in dual mate choice experiments, which associated with increased olfactory bulb-, but decreased in visual areas cell proliferation (optic tectum and *torus longitudinalis*) [41]. Thus, a complete view of adaptation to the photic changes at the organismal level will require integrating the contribution of the CMZ, spatial analyses across the neural retina, while also considering plastic changes beyond the retina.

Understanding how changes in environmental light are translated into progenitor or precursor cell activity may provide valuable insights into how neurogenic processes and progenitor cell behavior are regulated, especially when comparative approaches include species in which retinal regeneration is considered limited, as suggested for the medaka (Cab strain) due to restricted Müller glia potency [33]. Determining why the same environmental change recruits Müller glia in one species, but ONL precursors in another, could reveal how progenitor identity, activation, and cellular potential shape neurogenic responses, with broader implications for understanding the mechanisms that limit or enable regeneration.

## 5. Conclusions

Overall, our results show that retinal cell proliferation is modulated by changes in the light environment, with prolonged darkness recruiting distinct progenitor populations in *G. charrua* and *O. latipes*, two related teleost species with different natural habitats. These proliferative responses were followed by different changes in contrast sensitivity, highlighting adaptive neurogenesis as a component of visual responses to changing photic conditions. The differential recruitment of progenitor and precursor populations may reflect the ecological demands and evolutionary history of each species. Species-specific neurogenic responses in the retina may represent one of the mechanisms through which visual plasticity contributes to shaping evolutionary trajectories.

## Data Availability

The original contributions presented in this study are included in the article/Supplementary Material. Further inquiries can be directed to the corresponding authors.

## Supplementary Materials

The following supporting information can be downloaded at: https://www.mdpi.com/article/10.3390/cells15201886/s1, **Figure S1**: Optomotor response test scheme and analysis. (**a**) Experimental timeline. Darkness exposure timelines and OMR test (ai). Stimulus sequence for contrast sensitivity (aii) and spatial frequency (aiii) trials. (**b**) Semi-automated OMR stimulus timing analysis. Output of the manual annotation of the arena center and two reference crescents (arc 1, arc 2) used to calculate the frame intensity differences (bi). Plotted intensity differences calculated for the selected crescents, including the raw and smoothed values. Dotted lines indicate the thresholds to determine each moving phase. Color blocks denote the four directional stimulus stages (CW and CCW) (bii). (**c**) Bonsai rx workflow. Representative frame showing the segmentation steps of the test subject and its centroid (green dot, cii). (**d**) Schematic illustrating the response scores applied to classify optomotor responses for *G. charrua*. Abbreviations: LD: Light/dark photoperiod; DD: sustained darkness exposure; OMR: optomotor response, CW: clockwise, CCW: counterclockwise, RS: response score; **Figure S2**: Inter-species BrdU+ proliferative retinal cells after exposure to darkness in the *G. charrua* and *O. latipes* retina. (**a**) Schematic representation of the area depicted in b, and the experimental design for the darkness exposure groups. (**b**) Maximum intensity projections of the CMZ and dorsal neural retina depicting the BrdU+ nuclei for each species at different darkness exposure times (50 μm z-stack). Scale bars: 50 μm. (**c**) Number of BrdU+ nuclei per mm of retinal length for the ONL and INL of each species (*G. charrua*—n: 10/4, *O. latipes*—n: 12/5). (**d**) Number of EdU+ nuclei per mm of retinal length for for the ONL and INL of each species under darkness exposure periods (*G. charrua*—0 d n: 10/4; 14 d n: 12/4; 33 d n; 12/4, 45 d n: 15/5; *O. latipes*—0 d n: 12/5; 14 d n: 15/7; 33 d n: 12/5; 45 d n: 9/4). Box plots show median, and 25th and 75th percentiles, whiskers extend to the most extreme data points within 1.5 × IQR of the lower and upper quartiles. For each graph, sample numbers are expressed as the number of retinal sections/number of specimens. Significant differences were determined via Kruskal-Wallis and Dunn test (Bonferroni correction), significance is depicted as follows: * *p* < 0.05, ** *p* < 0.01, *** *p* < 0.001. Abbreviations: CMZ: Ciliary marginal zone, DD: Sustained darkness group, GCL: Ganglion cell layer, INL: Inner nuclear layer, LD: Control photoperiod group, NR: Neural retina, ONL: Outer nuclear layer; **Figure S3**: BrdU+ and EdU+ double labeled proliferative retinal cells after exposure to darkness in the *G. charrua* and *O. latipes* retina. (**a**) Schematic representation of the area depicted in b, and the experimental design for the darkness exposure groups. (**b**) Maximum intensity projections of the CMZ and dorsal neural retina depicting the BrdU+ nuclei for each species at different darkness exposure times (50 μm z-stack). Scale bars: 50 μm. (**c**) Number of BrdU+ nuclei per mm of retinal length for the ONL and INL of each species (*G. charrua*—n: 10/4, *O. latipes*—n: 12/5). (**d**) Number of EdU+ nuclei per mm of retinal length for for the ONL and INL of each species under darkness exposure periods (*G. charrua*—0 d n: 10/4; 14 d n: 12/4; 33 d n; 12/4, 45 d n: 15/5; *O. latipes*—0 d n: 12/5; 14 d n: 15/7; 33 d n: 12/5; 45 d n: 9/4). Box plots show median, and 25th and 75th percentiles, whiskers extend to the most extreme data points within 1.5 × IQR of the lower and upper quartiles. For each graph, sample numbers are expressed as the number of retinal sections/number of specimens. Significant differences were determined via Kruskal-Wallis and Dunn test (Bonferroni correction), significance is depicted as follows: * *p* < 0.05, ** *p* < 0.01, *** *p* < 0.001. Abbreviations: CMZ: Ciliary marginal zone, DD: Sustained darkness group, GCL: Ganglion cell layer, INL: Inner nuclear layer, LD: Control photoperiod group, NR: Neural retina, ONL: Outer nuclear layer; **Figure S4**: TUNEL+ nuclei after darkness in the *G. charrua* and *O. latipes* retina. (**a**) Schematic representation of the areas selected for TUNEL quantification. 100 μm wide confocal images (50 μm z-stack). The number of TUNEL+ nuclei was counted in the CMZ-adjacent neural retina and differentiated NR. Dorsal and ventral CMZ or NR were added per slice and compared between experimental groups. (**b**) CMZ adjacent sections depicting TUNEL stained nuclei (arrowheads) in the retina of *G. charrua* and *O. latipes*. Scale bars: 20 μm. (**c**–**j**) Violin plots with overlaid boxplots indicate TUNEL+ nuclei count in the CMZ-adjacent neural retinal (CMZ) and central neural retina (NR). (*G. charrua*—0 d n: 10/4; 14 d n: 11/4; 33 d n; 11/4, 45 d n: 12/4; *O. latipes*—0 d n: 11/5; 14 d n: 9/4; 33 d n: 9/5; 45 d n: 7/3). Box plots show median, and 25th and 75th percentiles, whiskers extend to the most extreme data points. Sample numbers are expressed as the number of retinal sections/number of specimens. Significant differences were determined via Kruskal-Wallis and Dunn test (Bonferroni correction). Significant *p*-values are indicated. Abbreviations: CMZ: Ciliary marginal zone, NR: Neural retina, ON: Optic nerve, GCL: Ganglion cell layer, INL: Inner nuclear layer, ONL: Outer nuclear layer; **Figure S5**: Retinal cell number across retinal regions and layers. DAPI-stained nuclei were quantified from in the neural retina adjacent to the CMZ (CMZ) and in the central retina (NR). For each region, nuclei were counted within 100-µm-wide areas in the dorsal and ventral retina, and the mean of both measurements was compared across darkness exposure conditions (see Figure S4a). Cell numbers are shown as nuclei per 100 µm of retinal width and classified according to the retinal layer. Within the ONL, cell types were further classified based on their position and nuclear morphology. Boxplots represent the distribution of individual values for each experimental group. (*G. charrua*—0 d n: 10/4; 14 d n: 11/4; 33 d n; 11/4, 45 d n: 11/4; *O. latipes*—0 d n: 11/4; 14 d n: 10/4; 33 d n: 10/4; 45 d n: 9/3). Box plots show median, and 25th and 75th percentiles, whiskers extend to the most extreme data points within 1.5 × IQR of the lower and upper quartiles. For each graph, sample numbers are expressed as the number of retinal sections/number of specimens. Differences were determined via Kruskal-Wallis and Dunn test (Bonferroni correction). Significant and tendencies *p*-values are indicated. Abbreviations: CMZ: ciliary marginal zone adjacent neural retina, NR: central neural retina, INL: Inner nuclear layer, ONL: Outer nuclear layer; **Figure S6**: Morphology of the *Garcialebias charrua* and *Oryzias latipes* adult neural retina. Confocal images of DAPI-stained retinal sections showing the organization of the major retinal layers in each species. Scale bars: 20 μm. GCL: Ganglion cell layer, INL: Inner nuclear layer; **Table S1**: Statistical comparisons of cell proliferation among darkness exposure conditions. BrdU, EdU or double labeled nuclei quantification for each retinal layer, for each species. Kruskal–Wallis test results are shown, followed by Dunn’s post hoc pairwise comparisons where applicable (Bonferroni correction). Significant responses are highlighted; **Video S1:** Example of positive responses to stimulus in spatial frequency test for *O. latipes*; **Video S2:** Example of non-stimulus-following behavior in contrast sensitivity test for *G. charrua*; **Video S3:** Example of positive responses to stimulus in contrast sensitivity test for *G. charrua*; **File S1:** Documentation and Use Guide for the semi-automated analysis pipeline of OMR assay; **File S2:** Coordinate defining Python script; **File S3**: Automatic Stimulus timing detection Python Script; **File S4:** OMR Data Analysis R Script.

## Abbreviations

The following abbreviations are used in this manuscript:

BrdU	5-bromo 2′-deoxyuridine
CMZ	Ciliary marginal zones
CPD	Cycles per degree
CW	Clock-wise
CCW	Counter-clock-wise
DD	Prolonged darkness
EdU	5-Ethynyl-2′-deoxyuridine
GCL	Ganglion cell layer
INL	Inner nuclear layer
IQR	Interquartile range
LD	Control photoperiod
NR	Neural retinal
OMR	Optomotor response
ONL	Outer nuclear layer

## References

[1] Stenkamp D.L. (2007). Neurogenesis in the Fish Retina. Int. Rev. Cytol..

[2] Fogg L.G., Isari S., Barnes J.E., Patel J.S., Marshall N., Salzburger W., de Busserolles F., Cortesi F. (2026). Deep-Sea Fish Reveal an Alternative Developmental Trajectory for Vertebrate Vision. Sci. Adv..

[3] Taylor S.M., Loew E.R., Grace M.S. (2015). Ontogenic Retinal Changes in Three Ecologically Distinct Elopomorph Fishes (Elopomorpha:Teleostei) Correlate with Light Environment and Behavior. Vis. Neurosci..

[4] Ausprey I.J. (2021). Adaptations to Light Contribute to the Ecological Niches and Evolution of the Terrestrial Avifauna. Proc. Biol. Sci..

[5] Carruthers M., Carleton K.L., Linderoth T., Bridle J., Hudson A.G., Malinsky M., Ndawala M.A., Ngatunga B.P., Saxon A.D., Shechonge A.H. (2025). Rapid Divergence of Visual Systems and Signaling Traits to Contrasting Light Regimes During Early Speciation of African Crater Lake Cichlid Fish. Mol. Biol. Evol..

[6] Härer A., Torres-Dowdall J., Meyer A. (2017). Rapid Adaptation to a Novel Light Environment: The Importance of Ontogeny and Phenotypic Plasticity in Shaping the Visual System of Nicaraguan Midas Cichlid Fish (*Amphilophus citrinellus* spp.). Mol. Ecol..

[7] Ehlman S.M., Sandkam B.A., Breden F., Sih A. (2015). Developmental Plasticity in Vision and Behavior May Help Guppies Overcome Increased Turbidity. J. Comp. Physiol. A-Neuroethol. Sens. Neural Behav. Physiol..

[8] Hartman E.J., Abrahams M.V. (2000). Sensory Compensation and the Detection of Predators: The Interaction between Chemical and Visual Information. Proc. Biol. Sci..

[9] Nieman C.L., Oppliger A.L., McElwain C.C., Gray S.M. (2018). Visual Detection Thresholds in Two Trophically Distinct Fishes Are Compromised in Algal Compared to Sedimentary Turbidity. Conserv. Physiol..

[10] Schulz E.J., Hoyer M.V., Canfield D.E. (1999). An Index of Biotic Integrity: A Test with Limnological and Fish Data from Sixty Florida Lakes. Trans. Am. Fish. Soc..

[11] Zhang Z., Fei F., Wang L., Rao Y., Li W., Gao X., Li A., Liu B. (2026). A Review of Adaptive Mechanisms in Fish Retinal Structure and Opsins Under Light Environment Regulation. Fishes.

[12] Crescitelli F., McFall-Ngai M., Horwitz J. (1985). The Visual Pigment Sensitivity Hypothesis: Further Evidence from Fishes of Varying Habitats. J. Comp. Physiol. A.

[13] Ebbesson L.O.E., Braithwaite V.A. (2012). Environmental Effects on Fish Neural Plasticity and Cognition. J. Fish Biol..

[14] Gomazkov O.A. (2014). Neurogenesis as an Adaptive Function of the Adult Brain. Biol. Bull. Rev..

[15] Lledo P.-M., Gheusi G. (2003). Olfactory Processing in a Changing Brain. NeuroReport.

[16] Lledo P.-M., Valley M. (2016). Adult Olfactory Bulb Neurogenesis. Cold Spring Harb. Perspect. Biol..

[17] Ortega-Perez I., Murray K., Lledo P.-M. (2007). The How and Why of Adult Neurogenesis. J. Mol. Histol..

[18] Zhao C., Deng W., Gage F.H. (2008). Mechanisms and Functional Implications of Adult Neurogenesis. Cell.

[19] Centanin L., Hoeckendorf B., Wittbrodt J. (2011). Fate Restriction and Multipotency in Retinal Stem Cells. Cell Stem Cell.

[20] Lindsey B.W., Hall Z.J., Heuzé A., Joly J.-S., Tropepe V., Kaslin J. (2018). The Role of Neuro-Epithelial-like and Radial-Glial Stem and Progenitor Cells in Development, Plasticity, and Repair. Prog. Neurobiol..

[21] Raymond P.A., Barthel L.K., Bernardos R.L., Perkowski J.J. (2006). Molecular Characterization of Retinal Stem Cells and Their Niches in Adult Zebrafish. BMC Dev. Biol..

[22] Bernardos R.L., Barthel L.K., Meyers J.R., Raymond P.A. (2007). Late-Stage Neuronal Progenitors in the Retina Are Radial Müller Glia That Function as Retinal Stem Cells. J. Neurosci..

[23] Lenkowski J.R., Raymond P.A. (2014). Müller Glia: Stem Cells for Generation and Regeneration of Retinal Neurons in Teleost Fish. Prog. Retin. Eye Res..

[24] Fischer A.J., Bosse J.L., El-Hodiri H.M. (2014). Reprint of: The Ciliary Marginal Zone (CMZ) in Development and Regeneration of the Vertebrate Eye. Exp. Eye Res..

[25] Miles A., Tropepe V. (2021). Retinal Stem Cell ‘Retirement Plans’: Growth, Regulation and Species Adaptations in the Retinal Ciliary Marginal Zone. Int. J. Mol. Sci..

[26] Bringmann A. (2001). Role of Muller Cells in Retinal Degenerations. Front. Biosci..

[27] Otteson D.C. (2017). Talkin’ about My (Re)Generation: The Who of Intrinsic Retinal Stem Cells. Neuroscience.

[28] Otteson D.C., D’Costa A.R., Hitchcock P.F. (2001). Putative Stem Cells and the Lineage of Rod Photoreceptors in the Mature Retina of the Goldfish. Dev. Biol..

[29] Johns P.R., Fernald R.D. (1981). Genesis of Rods in Teleost Fish Retina. Nature.

[30] Raymond P.A., Rivlin P.K. (1987). Germinal Cells in the Goldfish Retina That Produce Rod Photoreceptors. Dev. Biol..

[31] Sherpa R.D., Hui S.P. (2021). An Insight on Established Retinal Injury Mechanisms and Prevalent Retinal Stem Cell Activation Pathways in Vertebrate Models. Anim. Models Exp. Med..

[32] Chowdhury K., Lin S., Lai S.-L. (2022). Comparative Study in Zebrafish and Medaka Unravels the Mechanisms of Tissue Regeneration. Front. Ecol. Evol..

[33] Lust K., Wittbrodt J. (2018). Activating the Regenerative Potential of Müller Glia Cells in a Regeneration-Deficient Retina. eLife.

[34] Kodera K., Matsui H. (2022). Zebrafish, Medaka and Turquoise Killifish for Understanding Human Neurodegenerative/Neurodevelopmental Disorders. Int. J. Mol. Sci..

[35] Motamedi M., Akbarpour B. (2025). Regeneration in Fish, with Special Focus on Killifishes. Fish Physiol. Biochem..

[36] Alonso F., Terán G.E., Serra Alanís W.S., Calviño P., Montes M.M., García I.D., Barneche J.A., Almirón A., Ciotek L., Giorgis P. (2023). From the Mud to the Tree: Phylogeny of Austrolebias Killifishes, New Generic Structure and Description of a New Species (Cyprinodontiformes: Rivulidae). Zool. J. Linn. Soc..

[37] Berois N., Garcia G., de Sa R.O. (2015). Annual Fishes: Life History Strategy, Diversity, and Evolution.

[38] Loureiro M., de Sa R.O., Berois N., Garcia G., de Sa R.O. (2015). Diversity of Aplocheiloidei. Annual Fishes: Life History Strategy, Diversity, and Evolution.

[39] Casanova G., Rosillo J.C., Olivera-Bravo S., Fernández A.S., Berois N., Garcia G., de Sa R.O. (2015). Comparative Anatomy and Proliferative Zones of the Adult Austrolebias Brain. Annual Fishes: Life History Strategy, Diversity, and Evolution.

[40] Berrosteguieta I., Rosillo J.C., Herrera M.L., Olivera-Bravo S., Casanova G., Herranz-Pérez V., García-Verdugo J.M., Fernández A.S. (2022). Plasticity of Cell Proliferation in the Retina of *Austrolebias charrua* Fish under Light and Darkness Conditions. Curr. Res. Neurobiol..

[41] Berrosteguieta I., Herrera-Astorga L., Silva-Jaureguiberry S., Rosillo J.C., Passos C., Fernández A.S. (2026). Darkness Modifies Brain Cell Proliferation and Behavior in Female Austrolebias Reicherti Fish. J. Exp. Biol..

[42] Passos C., Tassino B., Rosenthal G.G., Reichard M., Berois N., Garcia G., de Sa R.O. (2015). Reproductive Behavior and Sexual Selection in Annual Fishes. Annual Fishes: Life History Strategy, Diversity, and Evolution.

[43] Herrera-Astorga L., Silva S., Berrosteguieta I., Rosillo J.C., Fernández A.S. (2025). Müller Glia in Short-Term Dark Adaptation of the *Austrolebias charrua* Retina: Cell Proliferation and Cytoarchitecture. Exp. Cell Res..

[44] Wittbrodt J., Shima A., Schartl M. (2002). Medaka—A Model Organism from the Far East. Nat. Rev. Genet..

[45] Inoue K., Takei Y. (2002). Diverse Adaptability in Oryzias Species to High Environmental Salinity. Zool. Sci..

[46] Naruse K., Loosli F., Ansai S., Birney E., Wittbrodt J. (2026). Medaka: A Novel Model for Analyzing Genome–Environment Interactions. Trends Genet..

[47] Hilgers L., Schwarzer J. (2019). The Untapped Potential of Medaka and Its Wild Relatives. eLife.

[48] Chuang H.-J., Chiu L., Yan J.-J., Chang C.-Y., Tang Y.-H., Chou M.-Y., Yu H.-T., Hwang P.-P. (2023). Responses of Medaka (*Oryzias latipes*) Ammonia Production and Excretion to Overcome Acidified Environments. J. Hazard. Mater..

[49] Fukuda S., Hiramatsu K., Mori M., Shikasyo S. (2005). An Adaptive Modeling Technique for Instream Fish Habitat Preference of Japanese Medaka (*Oryzias latipes*). J. Fac. Agric. Kyushu Univ..

[50] Papa N.G., Clivio G.A., Montagne J., Berois N., Garcia G., de Sa R.O. (2015). Husbandry, Reproduction, and Embryo Culture of Annual Fishes Under Laboratory Conditions. Annual Fishes: Life History Strategy, Diversity, and Evolution.

[51] Suzuki R., Kalšan D., Bhatti N., Pierotti S., Hofmann G., Birney E., Thumberger T., Wittbrodt J. (2026). A Scalable, High-Throughput Optomotor Response Pipeline for Quantitative Analysis of Vision Using Infinity-Pools. bioRxiv.

[52] Caves E.M., Troscianko J., Kelley L.A. (2020). A Customizable, Low-Cost Optomotor Apparatus: A Powerful Tool for Behaviourally Measuring Visual Capability. Methods Ecol. Evol..

[53] R Core Team (2024). R: A Language and Environment for Statistical Computing.

[54] Posit Team (2024). RStudio: Integrated Development Environment for R.

[55] Fernández A.S., Rosillo J.C., Casanova G., Olivera-Bravo S. (2011). Proliferation Zones in the Brain of Adult Fish Austrolebias (Cyprinodontiform: Rivulidae): A Comparative Study. Neuroscience.

[56] Hammer O., Harper D.A.T., Ryan P.D. (2001). PAST: Paleontological Statistics Software Package for Education and Data Analysis. Palaeontol. Electron..

[57] Schindelin J., Arganda-Carreras I., Frise E., Kaynig V., Longair M., Pietzsch T., Preibisch S., Rueden C., Saalfeld S., Schmid B. (2012). Fiji: An Open-Source Platform for Biological-Image Analysis. Nat. Methods.

[58] Luo L.-J., Jian H.-J., Harroun S.G., Lai J.-Y., Unnikrishnan B., Huang C.-C. (2021). Targeting Nanocomposites with Anti-Oxidative/Inflammatory/Angiogenic Activities for Synergistically Alleviating Macular Degeneration. Appl. Mater. Today.

[59] Herrera M.L., Silva S., Berrosteguieta I., Casanova G., Rosillo J.C., Fernández A.S. (2023). Rod Precursors in the Adult Retina of the *Austrolebias charrua* Annual Fish. Tissue Cell.

[60] Cobo R., Navarro-Sempere A., Segovia Y., García M. (2025). Adaptations of the Vertebrate Retina to Low-Light Conditions: A Review. Anat. Histol. Embryol..

[61] Rossi G.S., Labbé D., Wright P.A. (2022). Out of Water in the Dark: Plasticity in Visual Structures and Function in an Amphibious Fish. J. Exp. Zool. Part Ecol. Integr. Physiol..

[62] Lahne M., Piekos S.M., O’Neill J., Ackerman K.M., Hyde D.R. (2019). Photo-Regulation of Rod Precursor Cell Proliferation. Exp. Eye Res..

[63] Berois N., Arezo M.J., Papa N.G., Clivio G.A. (2012). Annual Fish: Developmental Adaptations for an Extreme Environment: Annual Fish. Wiley Interdiscip. Rev. Dev. Biol..

[64] Engeszer R.E., Patterson L.B., Rao A.A., Parichy D.M. (2007). Zebrafish in The Wild: A Review of Natural History And New Notes from The Field. Zebrafish.

[65] Parichy D.M. (2015). Advancing Biology through a Deeper Understanding of Zebrafish Ecology and Evolution. eLife.

