# Supplementary material for: Species-Specific Retinal Adaptation to Permanent Darkness in G. charrua and O. latipes
Source: Cells. 2026 Oct 15;15(20):1886. doi: 10.3390/cells15201886 (PMC13702896; doi:10.3390/cells15201886)
Supplement: Supplementary file 1 [file cells-15-01886-s001.zip › FileS1. 00_Analysis_Pipeline_Use_Guide.pdf]

This code was developed by Laura Herrera-Astorga as part of the analyses associated with Herrera-Astorga et al. (submitted) using ChatGPT 5.5 and 5.6.

This analysis pipeline uses Python for stimulus detection and preprocessing, followed by R for the behavioral analyses.

How to use:

After perform the OMR experiments to obtain video files. (.mp4 format is recommended) use the following pipeline for analysis.

1 Run `01_choose_arena_coordinate.py` to define visible stimulus regions. This first Python script is used to identify the portions of the visual stimulus that are visible in the experimental video. The script allows the user to define the visible crescent-shaped regions surrounding a circular central arena.
2 Run `02_Stripe_detection_frame_difference.py` to detect stimulus blocks. The second Python script identifies static and moving periods of the visual stimulus directly from the experimental video using the coordinates established by the previous script.
3 Run `03_OMR_Analysis.R`

STEP 1: Define the stimulus sampling region.

The analysis pipeline uses Python for stimulus detection for later analysis using R.

1. Requirements:

The Python scripts require the following packages:

```
opencv-python
numpy
pandas
matplotlib
```

A dedicated Python environment is recommended so that the required packages can be installed without interfering with other Python projects. The examples below use a Conda environment named `omr`. The `omr` environment should be activated whenever these Python scripts are used.

2. Before running a script, use the terminal to move to the folder containing the repository. We recommend keeping the Python scripts + videos in the same folder to avoid errors while indicating file names/paths.

3. Run the coordinate-selection script `01_choose_arena_coordinates.py`

4. The user will be prompted to indicate the desired file name to be analysed, and to define the position of the visible crescent-shaped regions by clicking five points: the center of the arena, the beginning and end of the first crescent, the beginning and end of the second crescent.

If needed for the analysis, adjust `arc_width_px` values.

5. The script will provide a parameter block containing values required by the next script, such as:

```
image_width_px = ...
image_height_px = ...
x0 = ...
y0 = ...
arc_radius_px = ...
arc_width_px = ...
visible_arc_ranges_deg = [...]
```

These coordinates will be exported as a .txt file and as graphical preview (.png) of the selected coordinates in the working directory. The entire block provided in the .txt file needs to be copied and pasted while running the next script.

Step 2— Detect stimulus timing

1. Run the stimulus-timing detection script `02_Stripe_detection_frame_difference.py`. During execution, the program will ask the user for several pieces of information.

2. Video path

The program will prompt the user to indicate the video to analyze `Video filename/path [video01.mp4]:`

If the video is located in the same folder as the script, only the filename is required, for example: `experiment01.mp4`

3. Output prefix

The script then asks for an output prefix `Output prefix [experiment01]:`

Pressing Enter keeps the video filename as the default prefix, otherwise type the desired prefix name. This prefix will be used for all files generated from that recording.

4. Stimulus-region coordinates

The user will then be prompted to indicate the coordinates (output generated by the previous script `01_choose_arena_coordinates.py`)

Paste the complete parameter block obtained in Step 1 (txt file) and then press Enter.

For example:

```
Suggested parameters for stimulus detection:
image_width_px = ...
image_height_px = ...
```

```
x0 = ...
y0 = ...
arc_radius_px = ...
arc_width_px = ...
visible_arc_ranges_deg = [...]
```

5. Stimulus direction

The program asks for the direction of the first moving stimulus phase:

`First moving subphase direction, CW or CCW [CW]:`
Use:

`CW` for clockwise movement, or:

`CCW` for counterclockwise movement.
If the experimental protocol alternates directions, the script can automatically assign consecutive phases as CW and CCW.

Please note, that if `costum` direction mode is chosen, the sequence will repeat cyclically.

6. Moving-phase duration

The program asks for the expected duration of each moving subphase: `Moving subphase duration in seconds [30.0].` By pressing Enter, the default 30s will be used.

7. Thresholding method

The frame-difference signal must be separated into static and moving periods.

The program provides two options: `Threshold method (otsu or manual):`

Enter: `otsu` to use automatic Otsu thresholding.

Enter: `manual` to define the threshold manually.

If manual thresholding is selected, the program asks:

`Manual motion threshold:` Enter the desired numerical value.

8. Width or spatial-frequency assignment

The detected moving phases can optionally be associated with the corresponding stimulus width or spatial-frequency values.

The available modes are:

```
none
per_moving
per_width_block
```

Pressing Enter automatically uses `none,` leaving this information empty.

`per_moving` assigns one supplied value to each moving subphase.

`per_width_block` assigns the same value to a specified number of consecutive moving subphases.

When values are entered, they should be separated by commas and written using decimal points.

For example: `0.2,0.4,0.6,0.8`

`min_motion_state_duration_s` Removes static/moving blocks shorter than the number entered (in seconds). Given the protocol design for this experiment, it is set to 8s. User should change values according to their own protocol design.

Please note, that the CW and CCW boundary are inferred by equal partitioning of the movement block.

9. Output

Two main diagnostic files should be inspected before proceeding:

`<prefix>_arc_sampling_preview.png`
This image shows the regions of the video used for stimulus detection. The marked arcs should coincide with the visible stimulus regions. If not, the arena coordinates should be redefined using the first script.

`<prefix>_motion_diagnostic.png`
This figure displays the frame-difference signal together with the threshold and the detected stimulus phases. The detected static and moving periods should correspond to the actual presentation of the visual stimulus in the video.

The stimulus-timing script generates:

```
<prefix>_arc_sampling_preview.png
<prefix>_motion_trace.csv
<prefix>_motion_blocks_detected.csv
<prefix>_phases_detected_protocol.csv
<prefix>_phases_for_R.csv
<prefix>_motion_diagnostic.png
<prefix>_parsed_crescent_parameters.txt
```

The main output required for the next steps in OMR analysis is: `<prefix>_phases_for_R.csv`

This table contains the timing and identity of the detected stimulus phases:

```
phase_id
start_s
end_s
direction
```

```
width
```

If no stimulus information was provided in step 8, manually include the widths used in the experiment for further analysis.

Step 3: OMR Behavioral Analysis – Visual acuity

`03_OMR_Analysis.R` performs batch analysis of optomotor response (OMR) tracking data from multiple specimens or videos. It combines centroid tracking data with the stimulus phase table generated by the preceding stimulus-timing workflow, calculates angular responses to stimulus motion, summarizes responses by stimulus level, estimates an individual acuity threshold, performs treatment comparisons, and exports quality-control tables and figures.

The analysis is designed for experiments in which each included manifest row represents one independent biological sample, normally one fish/video.

1. Requirements

   The script requires:

   - R
   - `tidyverse`
   - `rstatix`

   The analysis script checks that these packages are available and stops with an informative message if they are missing. It does not install packages automatically.

2. Set the project directory

   The script uses: `project_root <- getwd()`. Alternatively, replace `getwd() with` a full path.

   The working directory should contain: `experiment_manifest.csv`

3. Input files

   The analysis requires two files for each sample. These files are linked through `experiment_manifest.csv`.

   i. centroid tracking file;

      Each centroid file must be a CSV containing fish position coordinates for consecutive video frames. Accepted coordinate column names are `x, y` or `X, Y`. Missing x or y values are allowed. Intervals containing invalid positions are excluded from response calculations.

      The script treats the first row as the first frame and calculates time from the row number using: `fps_raw <- 30.`

   ii. a stimulus phase file (generated in STEP 2).

      Each phase file must contain:

**Column**	**Description**

start_s	Phase start time in seconds
end_s	Phase end time in seconds
direction	CW, CCW, static, or acclimation
width	Numeric stimulus-level value
phase_id	Optional; generated automatically if absent

Phase intervals must not overlap and every phase must satisfy end_s > start_s.

width is retained as the column name for compatibility with the stimulus-timing workflow, but the acuity calculation assumes that **larger numeric values represent harder stimuli**.

For spatial-frequency experiments, width should therefore normally contain the spatial frequency used for analysis, for example cycles per degree (cpd).

iii. The experiment manifest (experiment_manifest.csv. This file contains one row per sample to be analyzed.

Required columns

Column	Description
fish_id	Biological sample identifier
group_id	Treatment or experimental condition
centroid_path	Path to the centroid CSV
phase_path	Path to the phase CSV

Optional columns

Column	Description
include	Include/exclude the row. Accepted values include TRUE/FALSE, 1/0, yes/no. Blank = include.
sample_id	Unique analysis identifier. Defaults to fish_id.
trial_id	Used to distinguish duplicate sample identifiers when supplied.
output_dir	Custom output directory for that sample.
arena_x0	Arena center x-coordinate in tracking coordinates.
arena_y0	Arena center y-coordinate in tracking coordinates.
arena_radius_px	Arena radius in pixels. Recorded for quality-control purposes
min_radius_for_angle_px	Sample-specific central exclusion radius (in px). Overrides the default. If blank, default_min_radius_for_angle_px is used.

4. Analysis settings

Tracking: `fps_raw <- 30`. Frame rate of the centroid tracking data.

Arena center exclusion

```
default_min_radius_for_angle_px <- 10
```

Angular position becomes unstable when the fish is very close to the arena center. Positions inside this exclusion radius are therefore not used to calculate angular motion. By default, positions with a radial distance of less than 10 pixels from the arena center are excluded. A sample-specific value can be supplied in the manifest column `min_radius_for_angle_px`, which overrides the default for that sample.

Stimulus speed: `stim_speed_rad_s <- 0.31` Angular velocity of the moving visual stimulus in radians per second. This value must match the experimental stimulus.

Direction sign `fish_omega_sign <- 1` The default assumes that the tracking-coordinate convention and the stimulus direction convention produce positive response values when the fish moves with the stimulus.

Time binning `bin_size_s <- 3`. With this configuration, moving phases are divided into 3-s bins.

Bin response threshold `response_threshold <- 0.5` The response value is calculated as the fish net angular displacement divided by the angular displacement of the stimulus during the same bin. A value close to `1` therefore indicates net fish movement in the stimulus direction with an angular displacement similar to that of the stimulus. Valid bins with `response_value >= 0.5` are classified as `with_stimulus`; values `<= -0.5` are classified as `against_stimulus`; intermediate values are classified as `weak_or_none`.

Bin validity

```
min_bin_valid_fraction <- 0.75
min_bin_duration_fraction <- 0.50
```

These configurations indicate that a bin is used for response classification only when:

- it belongs to a CW or CCW phase;
- its duration is at least 50% of the nominal bin duration;
- at least 75% of its angular intervals are valid;
- the stimulus displacement is defined and non-zero.

With the default 3-s bins, the minimum accepted bin duration is 1.5 s.

Stimulus-level response threshold:
`fraction_with_stimulus_threshold_for_acuity <- 0.5`

A stimulus level is classified as responding when this fraction is at least `0.5`. For each fish and stimulus level, all valid CW and CCW bins are pooled. And the pooled response fraction is calculated.

```
number of bins classified with_stimulus
------------------------------------
       total number of valid bins
```

Minimum data per direction `min_valid_bins_per_direction_for_acuity <- 5`

Both CW and CCW must independently contain at least five valid response bins before the stimulus level is considered valid for acuity estimation. Here, 5 corresponds to half of the bins for each stimulus block. This prevents one direction from providing nearly all of the data even though CW and CCW are pooled for the final response fraction.

5. How the response is calculated: For each sample, the script performs the following steps.

i. Convert x/y position to angular position

Fish position is expressed relative to the arena center:

```
dx = x - arena_x0
dy = y - arena_y0
```

Angular position is then calculated around the arena center. Positions inside the central exclusion region are discarded for angular calculations.

ii. Unwrap the angle. Because angular coordinates normally jump between `+pi` and `-pi`, the angle is unwrapped over time so that continuous rotations can be measured correctly.

iii. Angular displacement is calculated between consecutive valid frames. The midpoint of each frame interval is used to assign that interval to an experimental phase.

iv. Divide moving phases into bins. Each phase is divided into bins of `bin_size_s`.

For each bin, the script calculates:

- net fish angular displacement;
- total absolute angular distance traveled;
- displacement in the stimulus direction;
- stimulus angular displacement;
- fraction of valid angular intervals.

v. Calculate the OMR response score For valid moving bins:

```
response_value =
    fish net angular displacement
    -----------------------------
    stimulus angular displacement
```

The stimulus sign is assigned so that movement with the stimulus produces a positive response.

vi. Each valid bin is classified as:

```
with_stimulus
against_stimulus
weak_or_none
```

Bins that fail the validity criteria are labeled: `not_valid`

vii. Pool CW and CCW at each stimulus level. For each fish and stimulus level, valid bins from both directions are pooled. The main acuity metric is: `fraction_with_stimulus_for_acuity`. CW and CCW are required to contain sufficient valid data separately, but individual bins contribute equally to the pooled fraction.

viii. Determine the acuity threshold. A stimulus level is responding when:

- both CW and CCW satisfy the minimum valid-bin requirement; and
- `fraction_with_stimulus_for_acuity` is greater than or equal to the configured fraction threshold.

The acuity threshold is then defined as: `highest stimulus value classified as responding`

6. Statistical analysis: comparisons at each stimulus level

Two fish-level metrics are compared among treatments separately at each stimulus value:

1. `fraction_with_stimulus_for_acuity`;
2. `pooled_mean_response_value`.

For each stimulus level the following test are performed: Kruskal-Wallis test compares treatments; Dunn pairwise comparisons, p values are adjusted using the method defined by: `multiple_testing_method <- "BH"`

Acuity threshold comparison

Fish with a detected acuity threshold are statistically compared among treatments using as described above. Fish for which no stimulus level met the response criterion do not contribute a numerical value to this threshold comparison.

7. Outputs

Combined outputs are written to: `group_analysis/`

Input and run records

```
manifest_used.csv
phase_expected_widths_by_sample.csv
batch_run_log.csv
R_session_info.txt
```

Combined response tables

```
combined_bin_response_values.csv
combined_phase_summary.csv
combined_width_direction_summary.csv
combined_width_summary.csv
combined_acuity_threshold_summary.csv
combined_qc_summary.csv
combined_width_plot_complete_all_widths.csv
final_fish_level_summary.csv
```

Descriptive summary

```
group_width_descriptive_summary.csv
```

Statistical outputs

```
stats_kw_fraction_with_stimulus_by_width.csv
stats_dunn_fraction_with_stimulus_by_width.csv
stats_kw_pooled_mean_response_by_width.csv
stats_dunn_pooled_mean_response_by_width.csv
stats_kw_acuity_width_threshold.csv
stats_dunn_acuity_width_threshold.csv
stats_acuity_detection_counts.csv
stats_fisher_acuity_detection.csv
```

Figures: The script generates five PDF figures:

`group_01_width_fraction_heatmap.pdf` Fish-by-stimulus heatmap of the pooled fraction of valid bins classified with the stimulus. `NT` indicates that the stimulus level was not tested. `NV` indicates that the stimulus level was tested but did not contain sufficient valid CW/CCW data for the acuity analysis.

`group_02_fraction_with_stimulus_by_width_boxplot.pdf` Fish-level pooled fraction of with-stimulus bins for each treatment and stimulus value. The dashed horizontal line indicates the stimulus-level response threshold.

`group_03_pooled_mean_response_by_width_boxplot.pdf` Bin-weighted mean OMR response score for each fish and stimulus value.

`group_04_acuity_width_threshold_boxplot.pdf` Highest responding stimulus value for fish with a detected acuity threshold.

`group_05_acuity_detection_fraction.pdf` Fraction of fish in each treatment for which an acuity threshold was detected.
