# Supplementary material for: Species-Specific Retinal Adaptation to Permanent Darkness in G. charrua and O. latipes
Source: Cells. 2026 Oct 15;15(20):1886. doi: 10.3390/cells15201886 (PMC13702896; doi:10.3390/cells15201886)
Supplement: Supplementary file 1 [file cells-15-01886-s001.zip › Table S1.pdf]

| | | *O. latipes* | | | | | | *G. charrua* | | | | | |
|---|---|---|---|---|---|---|---|---|---|---|---|---|---|
| | | | **ONL** | | | **INL** | | | **ONL** | | | **INL** | |
| | | **EdU** | **BrdU** | **EdU/BrdU** | **EdU** | **BrdU** | **EdU/BrdU** | **EdU** | **BrdU** | **EdU/BrdU** | **EdU** | **BrdU** | **EdU/BrdU** |
| **Kruskal-Wallis** | | | | | | | | | | | | | |
| H | | 17.78 | 17 | 20.58 | 11.75 | 9.93 | 10.2 | 5.877 | 5.318 | 5.736 | 17.78 | 4.336 | 2.741 |
| p | | **0.0005** | **0.00007** | **0.0001** | **0.008** | **0.019** | **0.016** | 0.1177 | 0.162 | 0.125 | **0.00048** | 0.227 | 0.4325 |
| η2 | | 1.399 | 0.318 | 0.4 | 0.199 | 0.158 | 0.164 | 0.064 | 0.052 | 0.61 | 1.399 | 0.03 | 0.006 |
| **Dunn's test with Bonferroni correction** | | | | | | | | | | | | | |
| 0 d | 14 d | **0.000012** | 0.75 | **0.002** | 1 | 1 | 1 | | | | 1 | | |
| 0 d | 33 d | 0.11 | 1 | 1 | 1 | 1 | 1 | | | | **0.0009163** | | |
| 0 d | 45 d | 1 | 0.064 | 1 | 0.107 | 0.18 | 0.106 | - | - | - | 1 | - | - |
| 14 d | 33 d | 0.14 | 1 | 0.1 | 0.636 | 1 | 0.997 | | | | **0.02** | | |
| 14 d | 45 d | **0.0000074** | **0.00026** | **0.00004** | **0.004** | 0.057 | **0.01** | | | | 1 | | |
| 33 d | 45 d | 0.0496 | **0.023** | 0.49 | 0.41 | **0.017** | 0.46 | | | | **0.004** | | |
